# Implementation of a hybrid neural network control technique to a cascaded MLI based SAPF

**DOI:** 10.1038/s41598-024-58137-0

**Published:** 2024-04-14

**Authors:** Rashmi Rekha Behera, Ashish Ranjan Dash, Satyasis Mishra, Anup Kumar Panda, Demissie Jobir Gelmecha

**Affiliations:** 1 Centurion University of Technology and Management, Paralakhemundi, Odisha India; 2 National Institute of Technology, Rourkela, Odisha India; 3 Adama Science and Technology University, Adama, Ethiopia

**Keywords:** Shunt Active Power Filter (SAPF), Cascaded MLI, Coupled transformer, Multilevel modulation, PS-PWM, Power quality, Harmonics, Artificial Neural Network (ANN), NBP based ${\varvec{i}}\cos \emptyset$ control, Energy science and technology, Engineering

## Abstract

This paper presents a naïve back propagation (NBP) based $i\cos \emptyset$ technique implemented to a cascaded multilevel inverter (MLI) based shunt active power filter (SAPF). The recommended control algorithm is applied to extract the fundamental component of load current and to decide the compensating current reference for harmonic elimination. The performance of the SAPF using the proposed NBP-based $i\cos \emptyset$ technique is compared with another two classical control techniques, such as, $i_{d} - i_{q}$ technique and $i\cos \emptyset$ technique. The accuracy of the proposed control technique depends on the tuned estimation of active and reactive weights. The performance study of the proposed SAPF with the proposed control technique is investigated under non-linear conditions, with balanced and unbalanced loading conditions. The results reveal that the recommended SAPF is efficient enough to reduce the harmonics from the source current with smooth variation in DC link voltage. The effectiveness of the proposed method is validated by simulation using MATLAB Simulink, and the real-time results are also validated by the experimental setup.

## Introduction

With a fabulous expansion in extremely nonlinear load implementations, the power grid system agonizes from various complications in power condition and quality. Expansion in renewable or inexhaustible energy resource applications also influences the quality of power. Generally, the power quality is mainly concerned with the condition of voltage and current. The voltage characteristic of the system entirely depends on the grid or supply voltage. Similarly, the current characteristic of the system entirely relies upon the load current. The low power quality consists of issues like voltage disparity in phases, harmonics in wave shape of voltage and current, swelling voltage, sagging voltage and flickering in voltage, imbalance in reactive power and also power blackout, etc. In the current situation, the active power filter (APF) is the most appropriate technique to deal with the issues regarding the quality of power. The implementation of APFs has been initiated to overcome the issues depicted before regarding the quality of electrical power^1^. There are various active power filters (APF) that are ready in the market for use in the quality improvement of electrical power; those are series APF^2,3^, shunt APF^4–6^ and unified power quality conditioner (UPQC)^7^. In the class of numerous APFs, shunt APFs are remarkably adequate during highly nonlinear loading conditions^8,9^. The SAPF is an effective device, which can inject current at PCC (point of common coupling) to recompense the harmonics in the supply current to make it sinusoidal.

Generally, the basic building blocks of a shunt APF are inverter (CSI/VSI), dc-link unit, control circuit, and inductive filter (discretionary). Out of the numerous types of SAPF, usually the APFs based on VSIs^7^ are more adopted due to reliability in nature. But, for high voltage applications, these classical VSIs are unable to deal with the device stress and losses. On the other hand the MLIs^10,11^ are capable to deal with high voltage applications with comparatively low switching frequency. Its harmonic minimization ability is also higher than traditional VSIs. As the classical MLIs are having a shortcoming of using a huge number of DC-link unit capacitors, which leads to an increase in the intricacy of the control system. Voltage balancing along with power-sharing becomes more problematic. And also, there will be a reduction in reliability as the capacitors count increases. With the increasing use of capacitors, it leads to more failure rate, as the capacitors are very venerable to faults^12^. This complication can be handled by reducing the requirement of capacitances in the DC link by using transformers in a cascaded manner. It can also produce a higher count of voltage steps by increasing the number of modules. With the application of transformers, the reliability increases due to a reduction in the number of capacitors in the system, and it also reduces the control problems. It contributes to the galvanic isolation of the grid system from the inverter.

This work approaches a novel MLI configuration^13^ which is cascaded through coupled transformer operating as a SAPF. This framework is based on traditional three-phase VSIs as the building blocks of the MLI. These voltage source inverter bridges are coupled with transformers, which are cascaded together to feed the grid system. This multilevel inverter can attain multiple levels of voltage steps with a minimum number of semiconductor switches as compared to other conventional MLIs. This multilevel voltage waveform can be achieved by implementing an appropriate modulation method and also appropriate turn ratios of the cascaded transformers.

The control system of a SAPF is very important for the effective operation of the SAPF connected to the grid system. Various control techniques and their applications are focused on the research area of SAPF. Few of them are p-q technique^14^, ($i_{d} - i_{q}$) method^15^, non-linear control methods, Lyapunov function-based method^16^, $i\cos \emptyset$ control method^17^ and many more. In last two decades, the artificial neural network (ANN) has significant progress in the research and application field. In^18^ an intelligent ANN-based swift harmonic detection system is described. In^19^ Hopfield ANN technique is analyzed for the detection of harmonics. Some more applications of ANN in the monitoring of harmonic distortion are explained in^20,21^. The major issue regarding the utilization of ANNs is picking up accurate dimensions and proper network topology. This turns into more complicated during training to very small error signals^22^. To overcome these complexities various mathematical replica is applied for one-step sizing, weight amendment, and normalization activity. It contributes to better response in an unsteady situation to control the SAPF in varying load conditions. Kernel incremental meta-learning process^23^ and ANN-based conductance estimation^24^ are implemented for a better solution for power quality issues. In^25,26^, EMD-based control strategy shows a real-time approach to reducing source current harmonics.

Out of various ANN techniques, the Naive back propagation (NBP) technique has a higher ability to treat the complex non-linear condition. The major advantages of this technique are self-organizing, adaptive learning, fault tolerance due to redundant information, and real-time implementation. In this work, detailed information of the estimation of the control technique followed by mathematical modeling is presented. The first layer of the ANN network is estimated by using the $i\cos \emptyset$ technique. By applying the sigmoidal criterion, the weighted components of load current for each phase are calculated. The initial weights for both outer and inner layers are applied. The weight correction is done by using the NBP method. Because of this, sizing adjustment, and adaptive learning function for the ANN are not necessary. There are various hybrid ANN techniques available in the literature^27–29,35,36^. Out of these NBP based $i\mathrm{cos\varnothing }$ control method is implemented for our proposed SAPF. This algorithm is very much useful for weighting factor extraction, and weight correction for the variable non-linear load current.

## Proposed SAPF configuration

**Figure 1:**
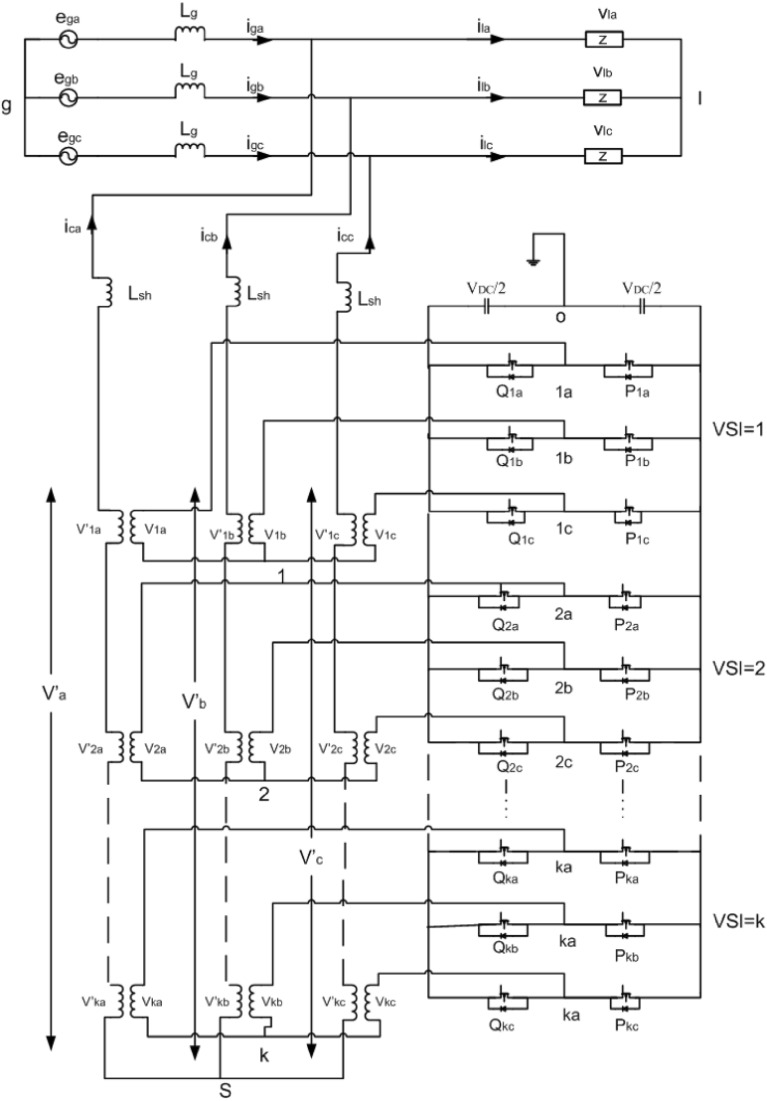
Suggested cascaded SAPF framework.

The proposed framework presented in Fig. 1 is consisting of an MLI, based on classical three-phase VSI bridges cascaded through coupled transformers, which feed the compensating current to the grid system. This MLI is coupled with the grid system at the common coupling position (PCC) as a SAPF. This suggested MLI topology can produce a higher count of voltage levels as compared to other classical topologies^30^. In this work a general framework is presented, where K numbers of VSI bridges are used. These bridges are cascaded through coupling transformers. Each VSI bridge with its coupled transformers can be considered as a single module. Each module is consisting of three legs for three different phases. Each leg has two switches ($p_{kj}$ and $q_{kj}$). The subscript ‘*k*’ represents the unit or cell number (*k* = 1, 2, 3…K), and similarly the subscript ‘*j*’ represents the phase name (*j* = a, b, c). So, there are six semiconductor devices in the three legs of each module. In total there are 6 K semiconductor devices in the suggested framework. The two switches in a leg are operated in a complementary state to one another to prevent short circuits of DC-link or uncertain voltage produced by the module. Therefore, exclusively eight possible switching states are possible for a single module. Therefore, the possible voltage levels per phase are $2v_{dc} /3$, $v_{dc} /3$, $- v_{dc} /3$ and $- 2v_{dc} /3$ which appear across the primary winding of the transformers, where $v_{dc}$ represents the voltage at the DC link. That is why we can get four voltage levels at the output of a single module.

The $v^{\prime}_{kj}$ is the secondary voltage of the coupled transformer.

1 $$ v^{\prime}_{kj} = { }N_{k} v_{kj} $$

So, the total resultant phase voltage of the cascaded secondary windings of the transformer per phase is $v^{\prime}_{j}$

2 $$ v^{\prime}_{j} = \mathop \sum \limits_{k = 1}^{K} v^{\prime}_{kj} $$

**Figure 2:**
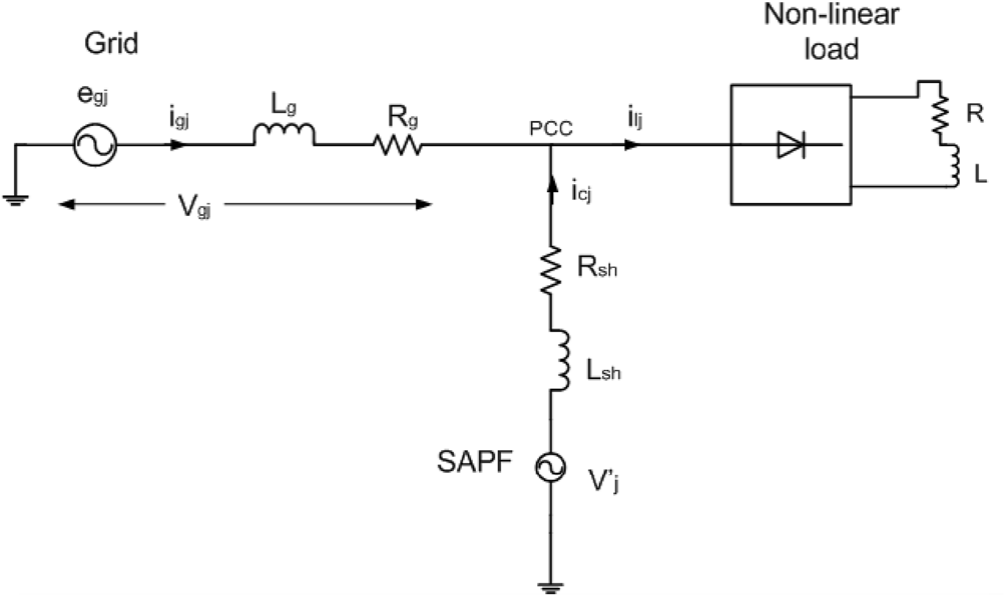
Single-line network diagram of the suggested SAPF coupled to grid.

Let the smoothing inductance is $l_{sh}$ at the PCC, and the equivalent resistance of SAPF is $r_{sh}$. Then,

3 $$ v^{\prime}_{j} - v_{gs} = \left[ {l_{sh} \frac{{di_{shj} }}{dt} + r_{sh} i_{shj} - l_{g} \frac{{di_{gj} }}{dt} - r_{g} i_{gj} + e_{gj} } \right]{ } $$

where, $v_{gs}$ is the voltage between the grid to the neutral point of the SAPF. $e_{gj}$ is the grid source voltage that is presented in Fig. 2. At PCC, the current equation applying KCL can be presented as

4 $$ i_{shj} = i_{lj} - i_{gj} $$

Putting the value of $i_{shj}$ from (4) in (3) we get

5 $$ v^{\prime}_{j} - v_{gs} = \left[ { - \left( {l_{g} + l_{sh} } \right)\frac{{di_{gj} }}{dt} - \left( {r_{g} + r_{sh} } \right)i_{gj} } \right] + \left[ {l_{sh} \frac{{di_{lj} }}{dt} + r_{sh} i_{lj} + e_{gj} } \right] $$

In (5) the second part of the right-hand side terms is the trepidation, which is to be recompensed by the SAPF (shunt active power filter). The resultant voltage of the transformer at the secondary winding is also denoted as

6 $$ v^{\prime}_{j} = { }v^{\prime}_{j0} - v^{\prime}_{0} $$

7 $$ v^{\prime}_{j0} = { }\mathop \sum \limits_{k = 1}^{K} N_{k} v_{kj0} $$

8 $$ v^{\prime}_{0} = \mathop \sum \limits_{k = 1}^{K} N_{k} v_{k0} = \mathop \sum \limits_{k = 1}^{K} \left( {\frac{{N_{k} }}{3}\mathop \sum \limits_{j} v_{kj0} } \right) $$

Now putting the value of $v^{\prime}_{0}$ from (8) into (6) we get

9 $$ v^{\prime}_{j} = { }v^{\prime}_{j0} - \left[ {\mathop \sum \limits_{k = 1}^{K} \left( {\frac{{N_{k} }}{3}\mathop \sum \limits_{j} v_{kj0} } \right)} \right] $$

We are able to develop a maximum 4Kcountof voltage levels in $v^{\prime}_{j}$ by considering the turns ratio of the transformers to be equal to one (i.e. $N_{k} = 1$). So, we can achieve a maximum of eight voltage steps in a two-module structure, and in that way, 12 voltage steps can be achieved by the three-module configuration of the MLI. Even, we can bring out a higher number of voltage steps in $v^{\prime}_{j}$ by allowing the turn ratios in a proper pattern for the transformers (i.e., $N_{k} = { }2^{k - 1}$). Taking the turn ratios in the proposed pattern we can achieve 12 voltage levels per phase in a two-module MLI, and twenty-eight voltage levels in a three-module MLI. We can get up to $2 \times \mathop \sum \limits_{k = 1}^{M} 2^{k}$ number of voltage steps by taking the turn ratios for the transformer in the pattern of $N_{k} = { }2^{k - 1}$.

## Reference compensating current generation

For precise execution of the SAPF, is most important that the reference current has to be generated correctly. The capacitance–voltage of the DC link performs a significant role in finding out the reference current during the nonlinear loading situation. Since the DC link provides the energy to load during any unwanted situation due to non-linear load implementation, the compensation requirement amount is determined by DC link voltage. An ANN controller is required to control this activity properly. DC voltage $v_{dc}$ should be maintained at a constant value to minimize the losses. The ANN is used to estimate the normalized weighting factor.

### NBP*-*based $i\cos \emptyset$ control

**Figure 3:**
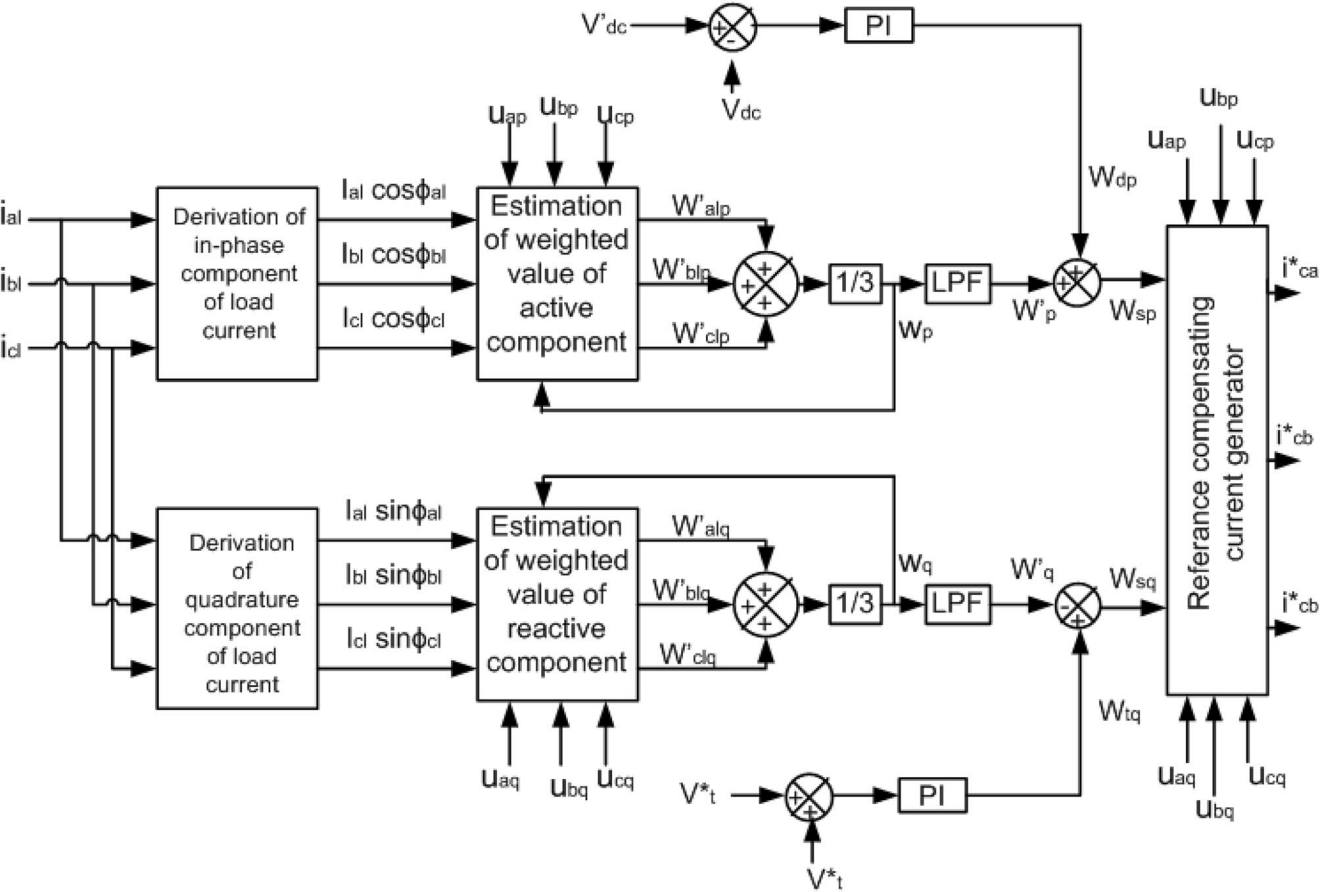
Block diagram of NBP based $i\mathrm{cos\varnothing }$ control technique.

The block diagram of this technique is presented in Fig. 3. At first, the load currents ($i_{al}$, $i_{bl}$, $i_{cl}$) and source voltages($v_{as}$, $v_{bs}$, $v_{cs}$) are sensed. Then the direct current components ($i_{al} \cos \emptyset_{al}$, $i_{bl} \cos \emptyset_{bl}$, $i_{cl} \cos \emptyset_{cl}$) and the quadrature current components ($i_{al} \sin \emptyset_{al} ,i_{bl} \sin \emptyset_{bl}$, $i_{cl} \sin \emptyset_{cl}$) are extracted by implementing $i\cos \emptyset$ technique^17–32^. After that these clustered weights are again processed through the NBP training mechanism. Here an updating of weight operation is done by an iterative method. As a result, a finely tuned weighted value of the active components ($w^{\prime}_{alp}$, $w^{\prime}_{blp}$, $w^{\prime}_{clp}$) and reactive components ($w^{\prime}_{alq}$, $w^{\prime}_{blq}$, $w^{\prime}_{clq}$) of the current is obtained. Then a filtered and tuned active component of weighting factor $w_{lp}$ and the reactive component of weighting factor $w_{lq}$ is generated. These weighting factors are not affected by noise, so, become more stable.

### Extraction of weighted values of active load current and reactive load current component

**Figure 4:**
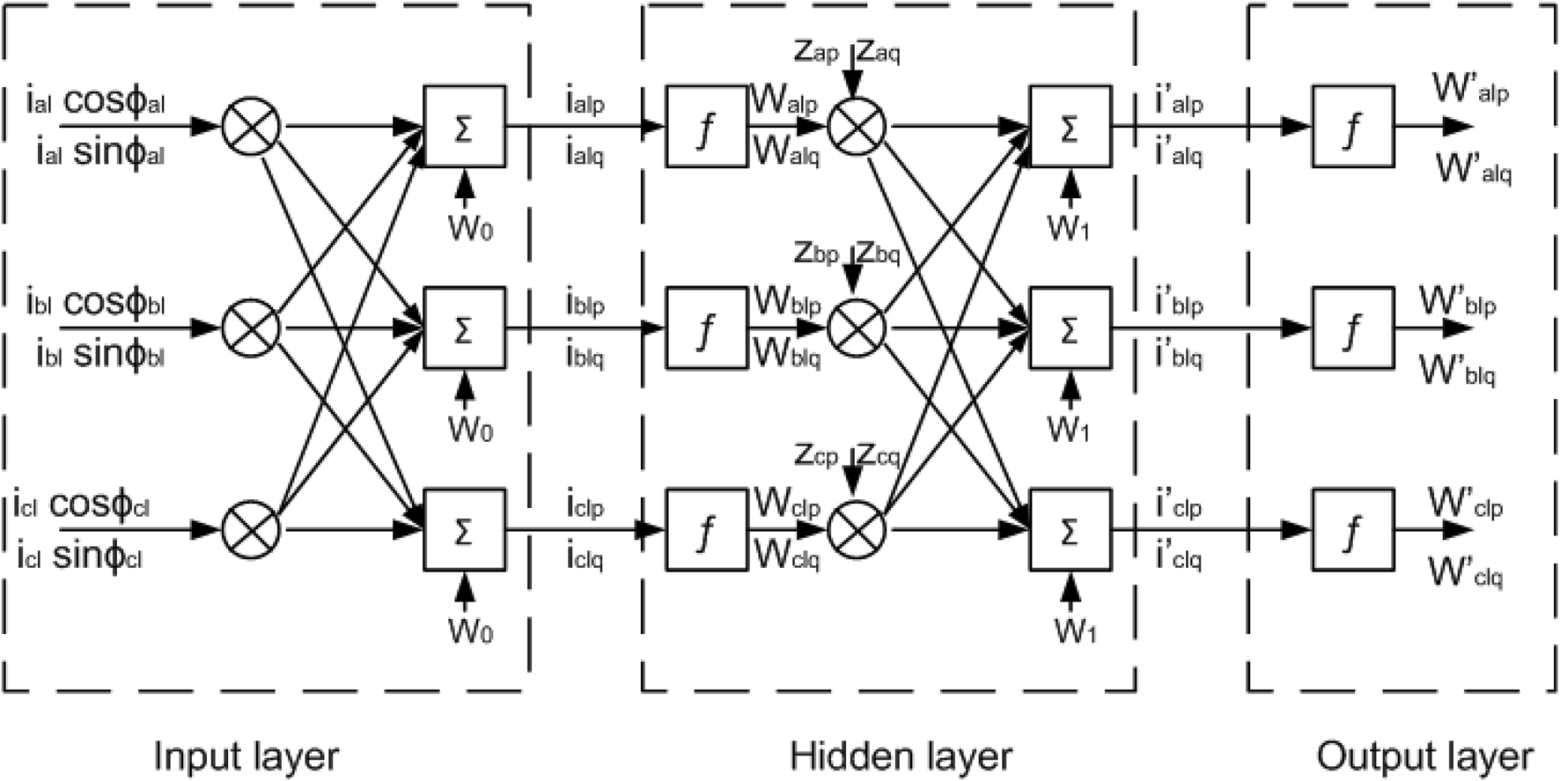
Extraction and updating weighted value using NBP technique.

The three-phase weights for the active components ($w^{\prime}_{alp}$, $w^{\prime}_{blp}$, $w^{\prime}_{clp}$) and reactive components ($w^{\prime}_{alq}$, $w^{\prime}_{blq}$, $w^{\prime}_{clq}$) are evaluated from the load current by NBP-based $i\cos \emptyset$ method. The extraction of weighted values and weight updating of the current components is presented in Fig. 4.

The weighted active components of current ($i_{alp}$, $i_{blp}$, $i_{clp}$) are generated as

10 $$ \left[ {\begin{array}{*{20}c} {i_{alp} } \\ {i_{blp} } \\ {i_{clp} } \\ \end{array} } \right] = w_{0} + \left[ {\begin{array}{*{20}c} {i_{al} \cos \emptyset_{al} } & {i_{bl} \cos \emptyset_{bl} } & {i_{cl} \cos \emptyset_{cl} } \\ \end{array} } \right]\left[ {\begin{array}{*{20}c} {u_{ap} } \\ {u_{bp} } \\ {u_{cp} } \\ \end{array} } \right] $$

where $w_{0}$ is the initial weight. $u_{ap}$, $u_{bp}$, $u_{cp}$ are unit voltage active components. The unit voltage active components are generated using the source voltages at PCC.

11 $$ \left[ {\begin{array}{*{20}c} {u_{ap} } \\ {u_{bp} } \\ {u_{cp} } \\ \end{array} } \right] = \frac{1}{{v_{t} }}\left[ {\begin{array}{*{20}c} {v_{as} } \\ {v_{bs} } \\ {v_{cs} } \\ \end{array} } \right] $$

where $v_{t}$ is the amplitude voltage evacuated as

12 $$ v_{t} = \sqrt {\frac{{2\left( {v_{as}^{2} + v_{bs}^{2} + v_{cs}^{2} } \right)}}{3}} $$

The active current component ($i_{alp}$, $i_{blp}$, $i_{clp}$) are operated through an uninterrupted sigmoid function to get weights ($w_{alp}$, $w_{blp}$, $w_{clp}$) as

13 $$ \left[ {\begin{array}{*{20}c} {w_{alp} } \\ {w_{blp} } \\ {w_{clp} } \\ \end{array} } \right] = \left[ {\begin{array}{*{20}c} {f\left( {i_{alp} } \right)} \\ {f\left( {i_{blp} } \right)} \\ {f\left( {i_{clp} } \right)} \\ \end{array} } \right] = 1 \div \left[ {\begin{array}{*{20}c} {1 + e^{{ - i_{alp} }} } \\ {1 + e^{{ - i_{blp} }} } \\ {1 + e^{{ - i_{clp} }} } \\ \end{array} } \right] $$

These weighted signals $w_{alp}$, $w_{blp}$, $w_{clp}$ are processed through hidden layers. The results of this hidden layer are $i^{\prime}_{alp}$, $i^{\prime}_{blp}$, $i^{\prime}_{clp}$, that are expressed as

14 $$ \left[ {\begin{array}{*{20}c} {i^{\prime}_{alp} } \\ {i^{\prime}_{blp} } \\ {i^{\prime}_{clp} } \\ \end{array} } \right] = w_{1} + \left[ {\begin{array}{*{20}c} {Z_{ap} } & {Z_{bp} } & {Z_{cp} } \\ \end{array} } \right]\left[ {\begin{array}{*{20}c} {w_{alp} } \\ {w_{blp} } \\ {w_{clp} } \\ \end{array} } \right] $$

where $w_{1}$ is the earliest weight of the invisible layer, and $Z_{ap}$, $Z_{bp}$, $Z_{cp}$ are revised weights of the active current component of each phase.

The revised weight signals $Z_{ap}$, $Z_{bp}$, $Z_{cp}$ can be extracted as given in (15). Where $w^{\prime}_{alp}$, $w^{\prime}_{blp}$, $w^{\prime}_{clp}$ are fundamental weights of the active components of load current. $w_{p}$ is the mean weight. $\mu$ is the rate of learning. In this work $\mu$ is selected as 0.6 within a range of 0–1. $f^{\prime}\left( {i^{\prime}_{alp} } \right)$, $f^{\prime}\left( {i^{\prime}_{blp} } \right)$ and $f^{\prime}\left( {i^{\prime}_{clp} } \right)$ are the first derivatives of $i^{\prime}_{alp}$, $i^{\prime}_{blp}$ and $i^{\prime}_{clp}$ respectively. $w_{alp}$, $w_{blp}$ and $w_{clp}$ are the outputs of the hidden layer.

15 $$ \left[ {\begin{array}{*{20}c} {Z_{ap} } \\ {Z_{bp} } \\ {Z_{cp} } \\ \end{array} } \right] = w_{p} + \left\{ {\mu \left[ {\begin{array}{*{20}c} {w_{p} - w^{\prime}_{alp} } & {w_{p} - w^{\prime}_{blp} } & {w_{p} - w^{\prime}_{clp} } \\ \end{array} } \right]\left[ {\begin{array}{*{20}c} {w_{alp} } \\ {w_{blp} } \\ {w_{clp} } \\ \end{array} } \right] + \left[ {\begin{array}{*{20}c} {f^{\prime}\left( {i^{\prime}_{alp} } \right)^{H} } \\ {f^{\prime}\left( {i^{\prime}_{blp} } \right)^{H} } \\ {f^{\prime}\left( {i^{\prime}_{clp} } \right)^{H} } \\ \end{array} } \right]} \right\}\delta $$

$i^{\prime}_{alp}$, $i^{\prime}_{blp}$ and $i^{\prime}_{clp}$ values are extracted and then processed through the sigmoid function to get $w^{\prime}_{alp}$, $w^{\prime}_{blp}$ and $w^{\prime}_{clp}$

16 $$ \left[ {\begin{array}{*{20}c} {w^{\prime}_{alp} } \\ {w^{\prime}_{blp} } \\ {w^{\prime}_{clp} } \\ \end{array} } \right] = \left[ {\begin{array}{*{20}c} {f\left( {i^{\prime}_{alp} } \right)} \\ {f\left( {i^{\prime}_{blp} } \right)} \\ {f\left( {i^{\prime}_{clp} } \right)} \\ \end{array} } \right] = \left[ {\begin{array}{*{20}c} {1 + e^{{ - i_{alp}^{\prime } }} } \\ {1 + e^{{ - i_{blp}^{\prime } }} } \\ {1 + e^{{ - i_{clp}^{\prime } }} } \\ \end{array} } \right] $$

The average weighted value $w_{p}$ is calculated by talking mean such as

17 $$ w_{p} = \frac{{w^{\prime}_{alp} + w^{\prime}_{blp} + w^{\prime}_{clp} }}{3} $$

Then it is processed through a LPF (low pass filter). The result of the LPF is $w^{\prime}_{p}$.

Similarly, the weighted reactive component current $i_{alq}$, $i_{blq}$ and $i_{clq}$ are generated as

18 $$ \left[ {\begin{array}{*{20}c} {i_{alq} } \\ {i_{blq} } \\ {i_{clq} } \\ \end{array} } \right] = w_{0} + \left[ {\begin{array}{*{20}c} {i_{al} \sin \emptyset_{al} } & {i_{bl} \sin \emptyset_{bl} } & {i_{cl} \sin \emptyset_{cl} } \\ \end{array} } \right]\left[ {\begin{array}{*{20}c} {u_{aq} } \\ {u_{bq} } \\ {u_{cq} } \\ \end{array} } \right] $$

where $u_{aq}$, $u_{bq}$ and $u_{cq}$ are unit voltage reactive component and can be derived as

19 $$ \left[ {\begin{array}{*{20}c} {u_{aq} } \\ {u_{bq} } \\ {u_{cq} } \\ \end{array} } \right] = \frac{1}{\sqrt 3 }\left[ {\begin{array}{*{20}c} 0 & {u_{bp} } & {u_{cp} } \\ {3u_{ap} } & {u_{bp} } & { - u_{cp} } \\ { - 3u_{ap} } & {3u_{bp} } & { - u_{cp} } \\ \end{array} } \right] $$

The reactive component of current $i_{alq}$, $i_{blq}$ and $i_{clq}$ are operated through uninterrupted sigmoid function to get $w_{alq}$, $w_{blq}$ and $w_{clq}$.

20 $$ \left[ {\begin{array}{*{20}c} {w_{alq} } \\ {w_{blq} } \\ {w_{clq} } \\ \end{array} } \right] = \left[ {\begin{array}{*{20}c} {f\left( {i_{alq} } \right)} \\ {f\left( {i_{blq} } \right)} \\ {f\left( {i_{clq} } \right)} \\ \end{array} } \right] = 1 \div \left[ {\begin{array}{*{20}c} {1 + e^{{ - i_{alq} }} } \\ {1 + e^{{ - i_{blq} }} } \\ {1 + e^{{ - i_{clq} }} } \\ \end{array} } \right] $$

These weighted signals $w_{alq}$, $w_{blq}$ and $w_{clq}$ are processed through hidden layers. The output of this hidden layer are $i^{\prime}_{alq}$, $i^{\prime}_{blq}$ and $i^{\prime}_{clq}$, such as

21 $$ \left[ {\begin{array}{*{20}c} {i^{\prime}_{alq} } \\ {i^{\prime}_{blq} } \\ {i^{\prime}_{clq} } \\ \end{array} } \right] = w_{1} + \left[ {\begin{array}{*{20}c} {Z_{aq} } & {Z_{bq} } & {Z_{cq} } \\ \end{array} } \right]\left[ {\begin{array}{*{20}c} {w_{alq} } \\ {w_{blq} } \\ {w_{clq} } \\ \end{array} } \right] $$

Here $Z_{aq}$, $Z_{bq}$ and $Z_{cq}$ are revised weights of the reactive current component of each phase and can be represented as shown in (22). where $w^{\prime}_{alq}$, $w^{\prime}_{blq}$ and $w^{\prime}_{clq}$ are fundamental weighted amplitudes of reactive load current components. $w_{q}$ is the mean weight. $\mu$ is the rate of learning. $f^{\prime}\left( {i^{\prime}_{alq} } \right)$, $f^{\prime}\left( {i^{\prime}_{blq} } \right)$ and $f^{\prime}\left( {i^{\prime}_{clq} } \right)$ are the first derivatives of $i^{\prime}_{alq}$, $i^{\prime}_{blq}$ and $i^{\prime}_{clq}$ respectively. $w_{alq}$, $w_{blq}$ and $w_{clq}$ are the outputs of the hidden layer.

22 $$ \left[ {\begin{array}{*{20}c} {Z_{aq} } \\ {Z_{bq} } \\ {Z_{cq} } \\ \end{array} } \right] = w_{q} + \left\{ {\mu \left[ {\begin{array}{*{20}c} {w_{q} - w^{\prime}_{alq} } & {w_{q} - w^{\prime}_{blq} } & {w_{q} - w^{\prime}_{clq} } \\ \end{array} } \right]\left[ {\begin{array}{*{20}c} {w_{alq} } \\ {w_{blq} } \\ {w_{clq} } \\ \end{array} } \right] + \left[ {\begin{array}{*{20}c} {f^{\prime}\left( {i^{\prime}_{alq} } \right)^{H} } \\ {f^{\prime}\left( {i^{\prime}_{blq} } \right)^{H} } \\ {f^{\prime}\left( {i^{\prime}_{clq} } \right)^{H} } \\ \end{array} } \right]} \right\}\delta $$

The values $i^{\prime}_{alq}$, $i^{\prime}_{blq}$ and $i^{\prime}_{clq}$ are extracted and processed through the uninterrupted sigmoidal function to generate the weighted signals $w^{\prime}_{alq}$, $w^{\prime}_{blq}$ and $w^{\prime}_{clq}$, such as

23 $$ \left[ {\begin{array}{*{20}c} {w^{\prime}_{alq} } \\ {w^{\prime}_{blq} } \\ {w^{\prime}_{clq} } \\ \end{array} } \right] = \left[ {\begin{array}{*{20}c} {f\left( {i^{\prime}_{alq} } \right)} \\ {f\left( {i^{\prime}_{blq} } \right)} \\ {f\left( {i^{\prime}_{clq} } \right)} \\ \end{array} } \right] = \left[ {\begin{array}{*{20}c} {1 + e^{{ - i_{alq}^{\prime } }} } \\ {1 + e^{{ - i_{blq}^{\prime } }} } \\ {1 + e^{{ - i_{clq}^{\prime } }} } \\ \end{array} } \right] $$

The average weighted value of $w_{q}$ is calculated by talking mean, such as

24 $$ w_{q} = \frac{{w\prime_{alq} + w\prime_{blq} + w\prime_{clq} }}{3} $$

Then it is processed through a LPF. The output of the LPF is $w\prime_{q}$.

### Evaluation of active reference component

The error in DC voltage $v_{dc}$ can be evaluated by taking the difference of sensed DC voltage $v_{dc}$ from DC reference voltage $v_{dc}^{*}$. This error signal is processed through PI controller, such as

25 $$ w_{dp} = k_{dp} v_{dc} + k_{ip} \smallint v_{dc} dt $$

The active weighted reference current component $w_{sp}$ is obtained by adding the average magnitude of active load current component $w^{\prime}_{p}$ and the output of the PI controller $w_{dc}$.

26 $$ w_{sp} = w_{dp} + w^{\prime}_{p} $$

### Evaluation of active reference component

The error signal in AC voltage can be evaluated by taking the difference of sensed AC voltage $v_{t}$ from AC reference voltage $v_{t}^{*}$. This error signal is processed through PI controller, such as

27 $$ w_{tq} = k_{tq} v_{t} + k_{iq} \smallint v_{t} dt $$

The reactive weighted reference current component $w_{sq}$ is derived by subtracting the mean magnitude of reactive load component $w^{\prime}_{q}$ from the output result of PI controller $w_{tq}$.

28 $$ w_{sq} = w_{tq} - w^{\prime}_{q} $$

### Evaluation of reference compensating current components

The three-phase active reference current components $i_{cap}$, $i_{cbp}$ and $i_{ccp}$ are predicted by multiplying matrix of unit voltage active components $u_{ap}$, $u_{bp}$, $u_{cp}$ with the total active weighted current component $w_{sp}$.

29 $$ \left[ {\begin{array}{*{20}c} {i_{cap} } \\ {i_{cbp} } \\ {i_{ccp} } \\ \end{array} } \right] = w_{sp} \left[ {\begin{array}{*{20}c} {u_{ap} } \\ {u_{bp} } \\ {u_{cp} } \\ \end{array} } \right] $$

Similarly, the three-phase reactive reference current components $i_{caq}$, $i_{cbq}$ and $i_{ccq}$ are estimated by multiplying unit voltage reactive components $u_{aq}$, $u_{bq}$, $u_{cq}$ matrix with the total reactive weighted current component $w_{sq}$.

30 $$ \left[ {\begin{array}{*{20}c} {i_{caq} } \\ {i_{cbq} } \\ {i_{ccq} } \\ \end{array} } \right] = w_{sq} \left[ {\begin{array}{*{20}c} {u_{aq} } \\ {u_{bq} } \\ {u_{cq} } \\ \end{array} } \right] $$

The reference compensating current $i_{ca}^{*}$, $i_{cb}^{*}$ and $i_{cc}^{*}$ are realized by adding these active and reactive components of reference current, such as

31 $$ \left[ {\begin{array}{*{20}c} {i_{ca}^{*} } \\ {i_{cb}^{*} } \\ {i_{cc}^{*} } \\ \end{array} } \right] = \left[ {\begin{array}{*{20}c} {i_{cap} } \\ {i_{cbp} } \\ {i_{ccp} } \\ \end{array} } \right] + \left[ {\begin{array}{*{20}c} {i_{caq} } \\ {i_{cbq} } \\ {i_{ccq} } \\ \end{array} } \right] $$

Then the reference current for compensation ($i_{ca}^{*}$, $i_{cb}^{*}$, $i_{cc}^{*}$) are compared with the compensating current. The result of this comparison generates signal for modulation to produce the firing pulses for the semiconductor devices of the MLI.

## Generation of triggering signals for the semiconductor switches

**Figure 5:**
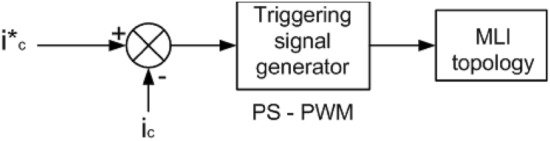
Block Diagram for the PWM Technique application.

**Figure 6:**
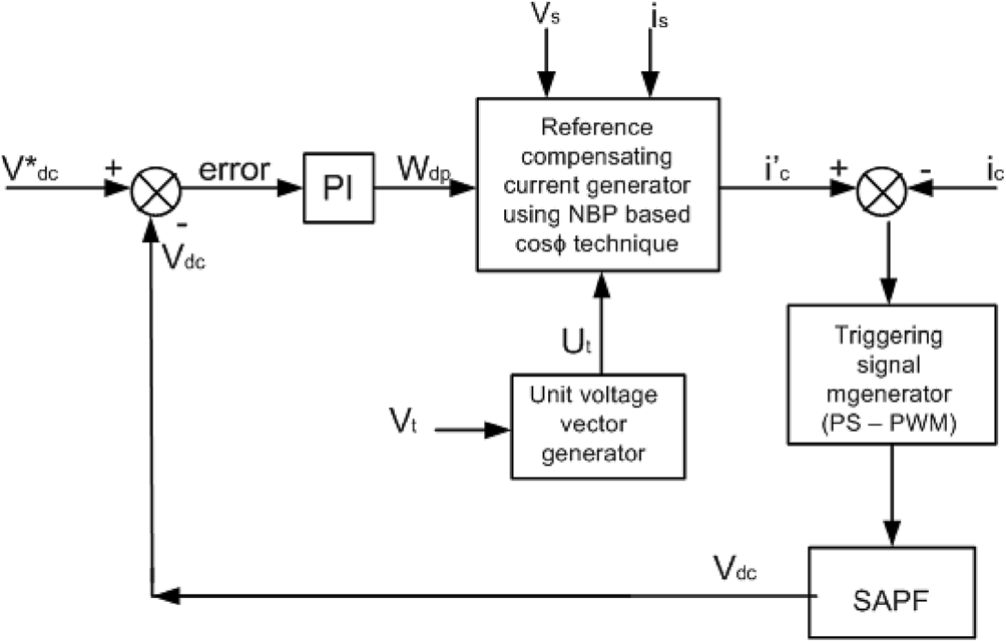
Control strategy block diagram for the SAPF.

In the present work the multi-carrier based PWM, called phase-shifted pulse width modulation (PS-PWM) is applied^33,34^. The reference current for compensation ($i_{ca}^{*}$, $i_{cb}^{*}$, $i_{cc}^{*}$) are correlated here with the compensation currents of the SAPF (*i*_*ca*_*, i*_*cb*_*, i*_*cc*_). Now the difference is provided to the PWM block to produce the firing pulse signals for the semiconductor devices of the MLI, where the deference of the compensating current to the reference current is compared with triangular carrier waves. There is a 3* K* number of carriers required, and these carriers are shifted by phase to develop firing pulses for each switch in the MLI. The block diagram of the PWM technique is presented in Fig. 5. Because of this, the power-sharing between the modules or cells becomes systematic. The diagram in Fig. 6 represents the close loop control strategy of the SAPF. In this diagram the functioning of the total control system is represented including the ANN (NBP-based $i\cos \emptyset$ technique) based reference current generator, triggering pulse generator and the SAPF as a whole.

## Results of simulation

**Figure 7:**
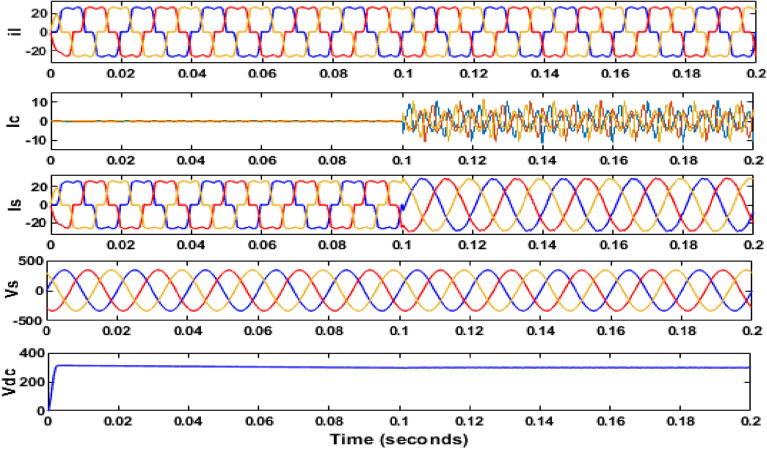
Operating characteristic of proposed SAPF during non-linear loading condition.

**Figure 8:**
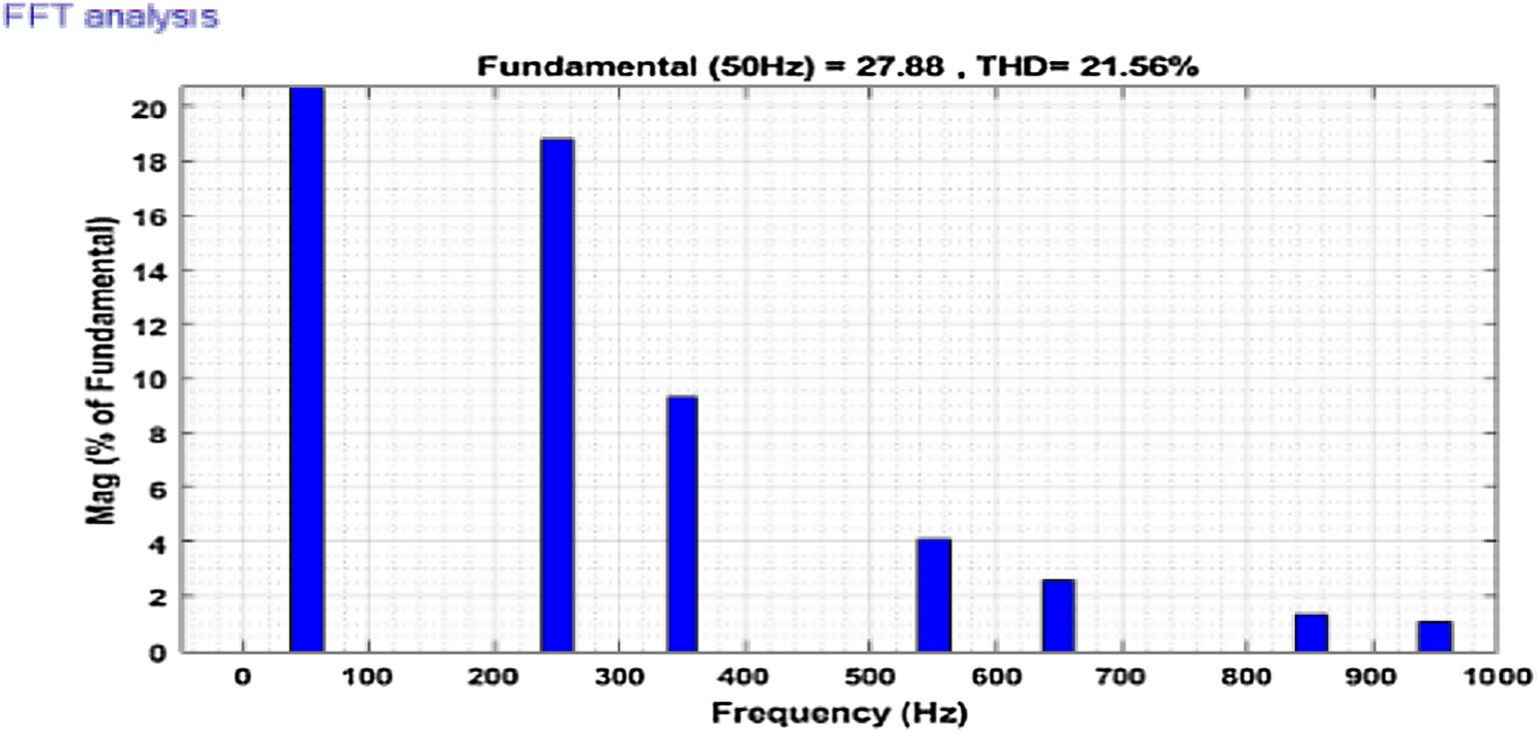
THD of grid current ‘$i_{s}$’ before compensation in nonlinear loading condition.

**Figure 9:**
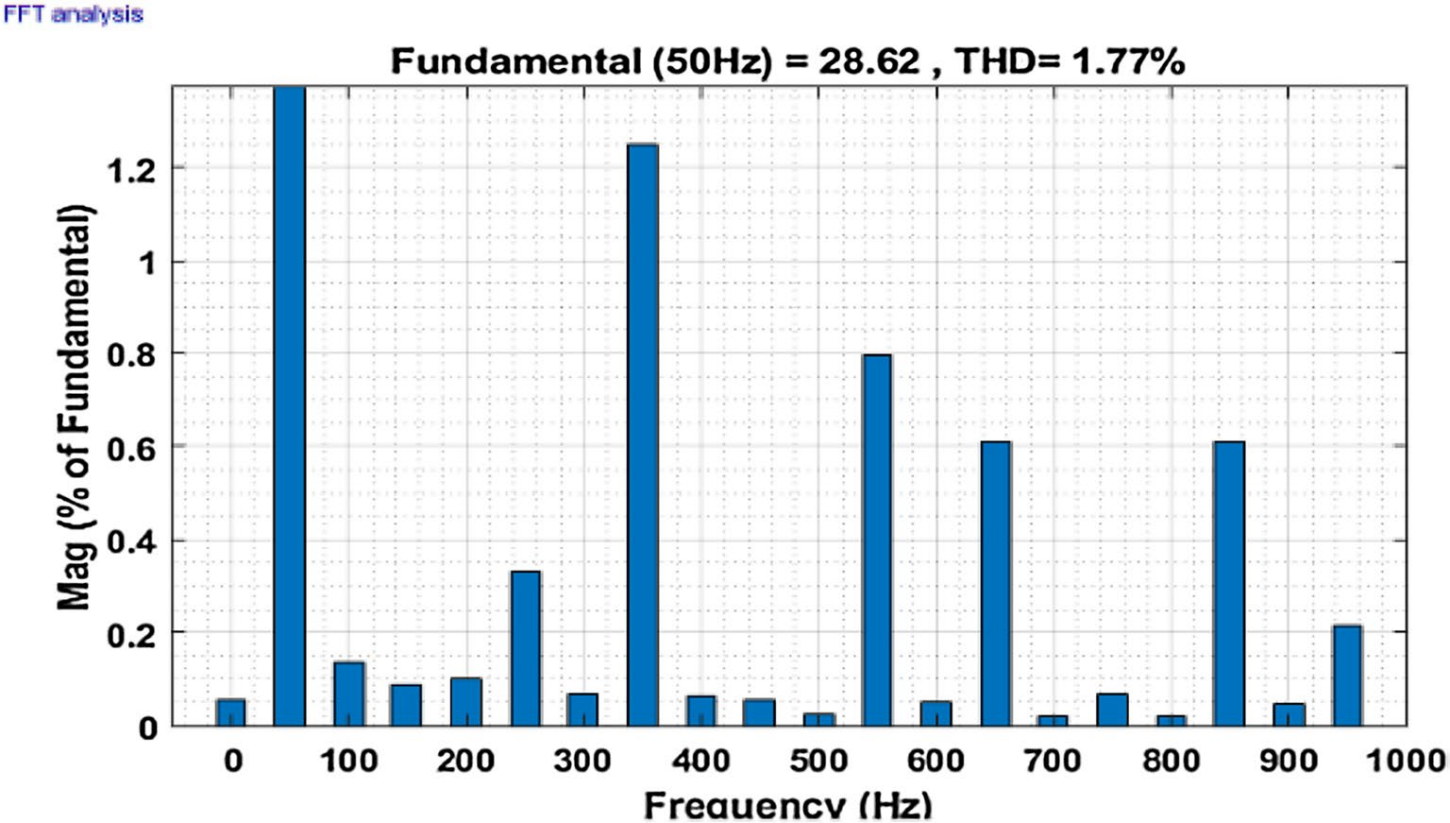
THD of grid current ‘$i_{s}$’ after compensation in nonlinear loading condition.

The simulation model for the SAPF with different control strategies is designed in MATLAB/SIMULINK using the power system toolbox in it. The proposed SAPF is investigated under various load conditions. During the observation, the grid voltage is kept ideal. The parameter specification is presented in Table 1. In the simulation model, a nonlinear load is considered to be connected to the grid. This nonlinear load is consisting of an unrestrained rectifier bridge with an inductive load. The magnitude of resistance R is equal to 20Ω and that of the inductance L is equal to 20 mH. The SAPF is introduced after 0.1 s. Figure 7 shows the working behaviour of the active filter under this balanced non-linear loading condition. The load current $i_{l}$, compensating current $i_{c}$, source current $i_{s}$, source voltage $v_{s}$, and DC link voltage $v_{dc}$ are presented respectively. The THD of the grid source current is realized to be 21.56% before the initialization of the active filter as shown in Fig. 8. But, it reduced to 1.77% after the initialization of the SAPF as shown in Fig. 9.

**Table 1:** Parameter specification for simulation.

Sl. no.	System parameters	Specification
1	Grid voltage ‘$V_{s}$’ [line–line]	415 V
2	Switching frequency ‘$f_{s}$’	50 Hz
3	DC link capacitance ‘$C_{dc}$’	2200 μF
4	Non-linear load [first]	Uncontrolled rectifier bridge with RL load R = 20 Ω and L = 20 mH
5	Non-linear load [second]	Uncontrolled rectifier bridge with RL load R = 25 Ω and L = 25 mH
6	Unbalanced 3-phase RL load	${R}_{1}$= 30 Ω, ${L}_{1}$= 10 mH ${R}_{2}$= 10 Ω, ${L}_{2}$= 20 mH ${R}_{3}$= 20 Ω, ${L}_{3}$= 20 mH
7	Single-phase RL load	R = 30 Ω, L = 20 mH

**Figure 10:**
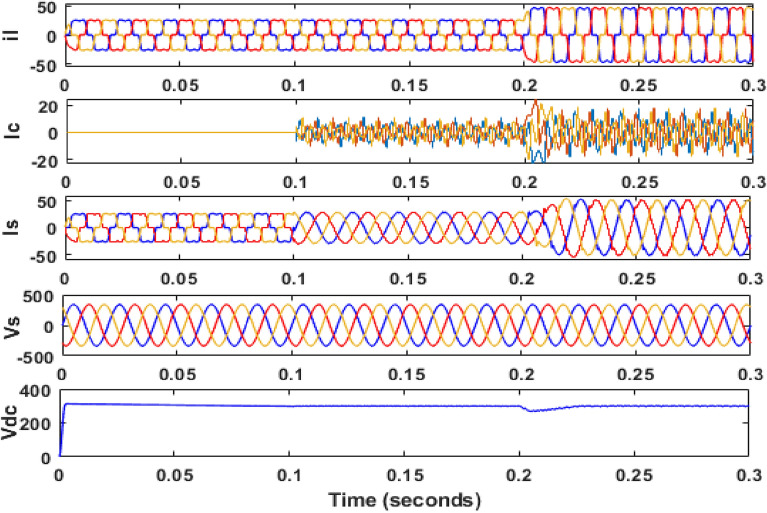
Performance characteristic of proposed SAPF with a step changing non-linear load.

**Figure 11:**
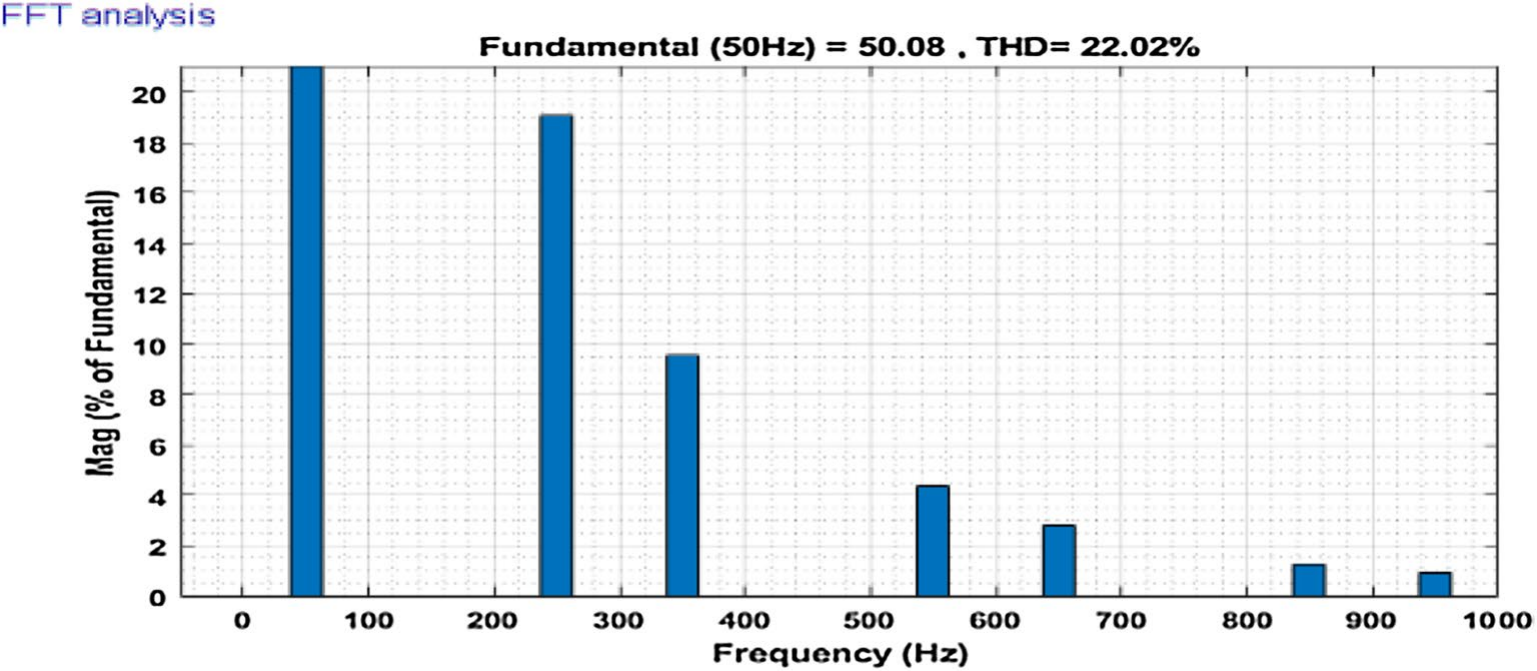
THD of grid current ‘$i_{s}$’ before compensation during step changed non-linear load.

**Figure 12:**
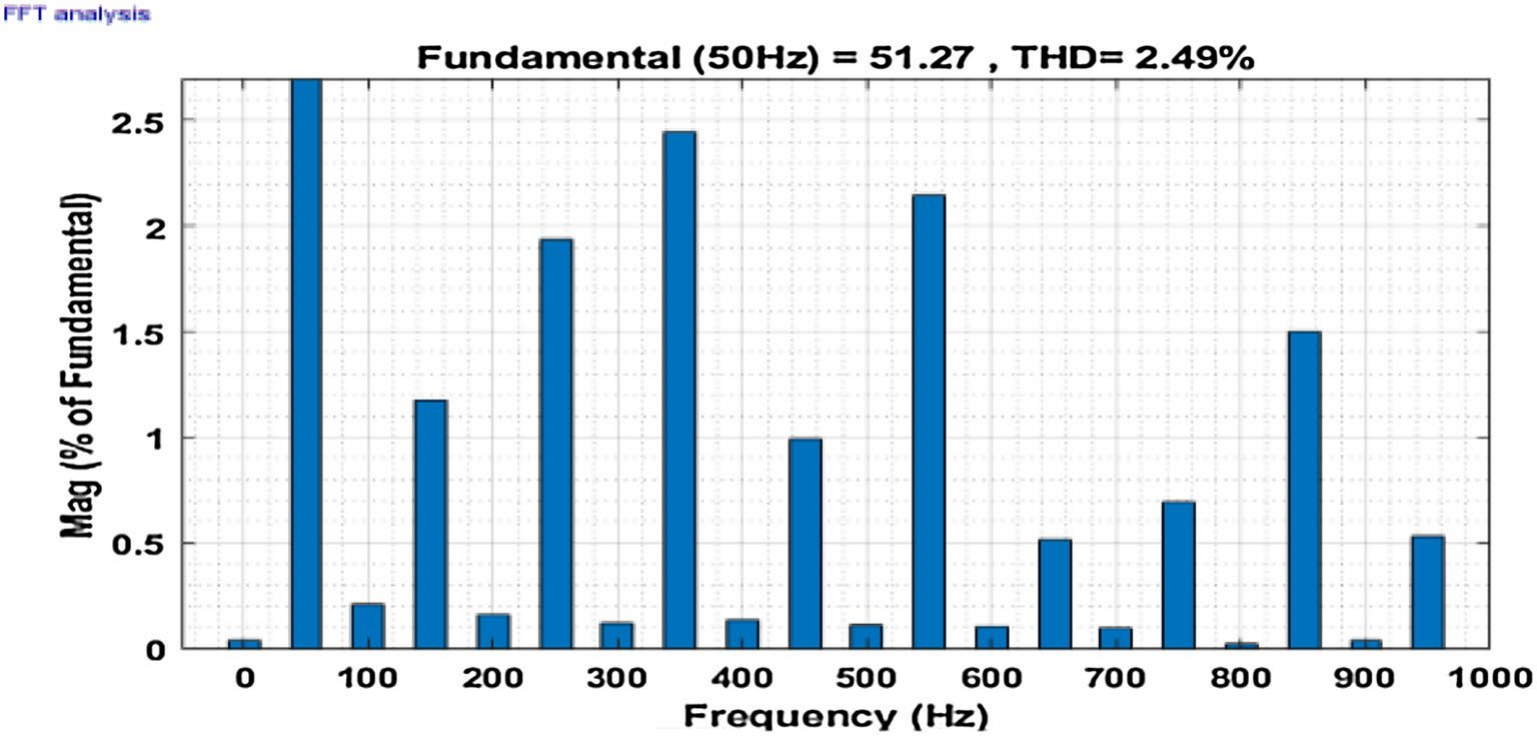
THD of grid current ‘$i_{s}$’ after compensation during step changed non-linear load.

**Figure 13:**
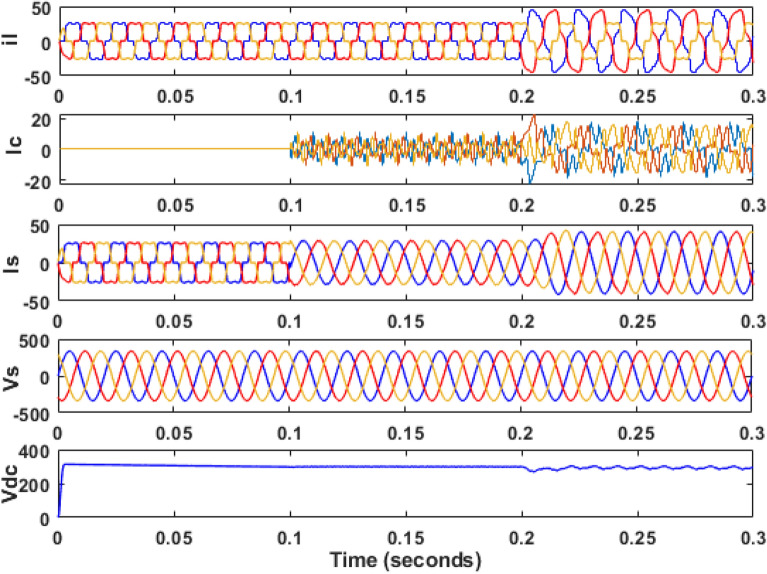
Performance characteristic of proposed SAPF with addition of a single-phase inductive load in between line ‘a’ and ‘b’.

**Figure 14:**
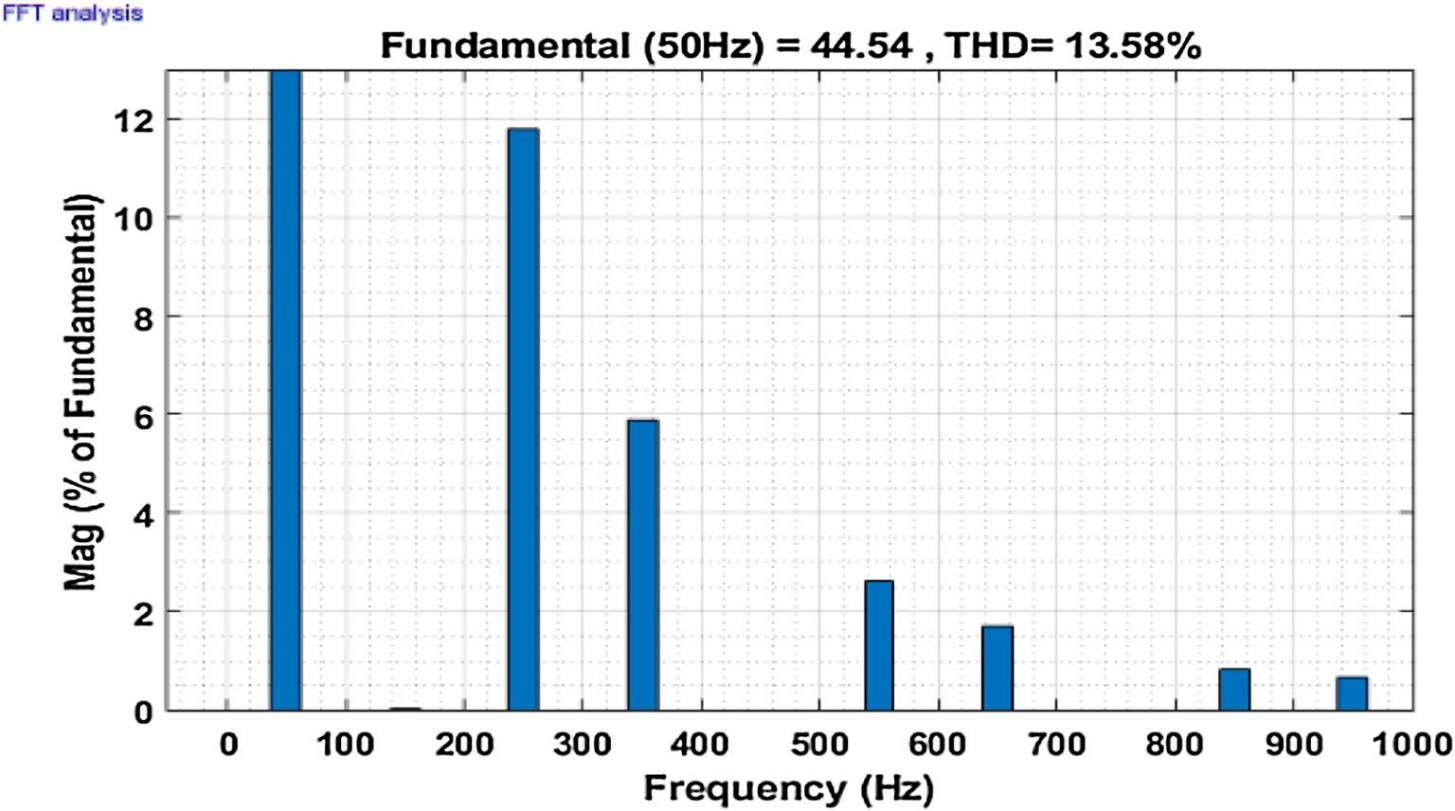
THD of grid current ‘$i_{s}$’ before compensation with addition of a single-phase inductive load in between line ‘a’ and ‘b’.

**Figure 15:**
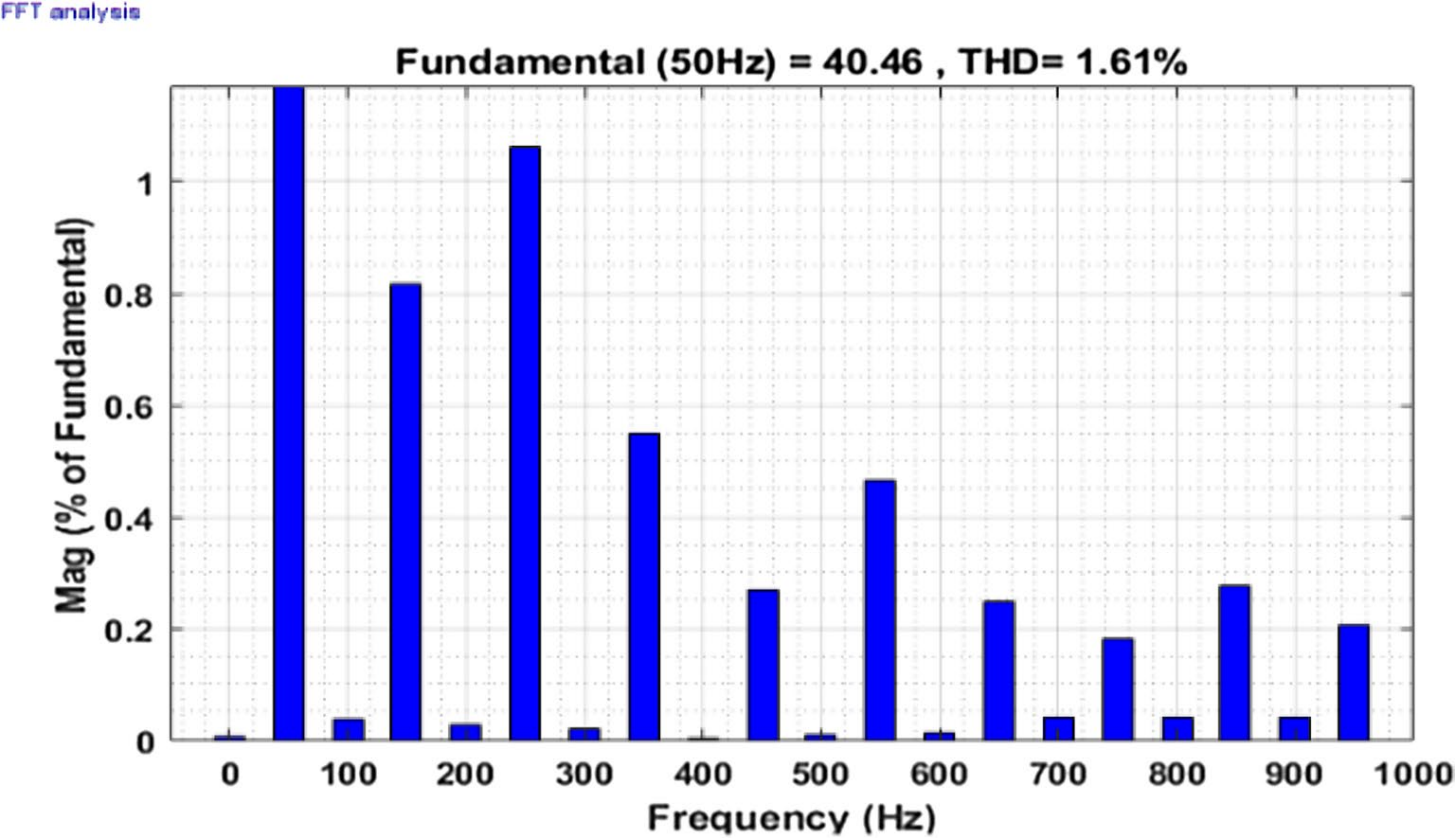
THD of grid current ‘$i_{s}$’ after compensation with addition of a single-phase inductive load in between line ‘a’ and ‘b’.

Then the system performance is observed under a variable loading condition. After 0.2 s a step variation is done in the load. A balanced non-linear load is added parallel to the present load. The observation result is revealed in Fig. 10. The THD value in the grid source current without filtering is realized to be 22.02% as shown in Fig. 11. It reduced to 2.49% after filtering as depicted in Fig. 12. After that the system performance is observed for an unbalanced loading condition. Here a single-phase inductive RL load is connected between two phases, those are phase ‘a’ and phase ‘b’, where R = 30Ω and L = 20 mH. Here the active filter is initiated at 0.1 s and the variation in load is done at 0.2 s. The performance behavior is shown in the Fig. 13. Figures 14 and 15 show the FFT result of the unbalanced loading situation before and after filtering respectively. It is found the before filtering the THD value was 13.58%, which reduces to 1.61% after filtering.

**Figure 16:**
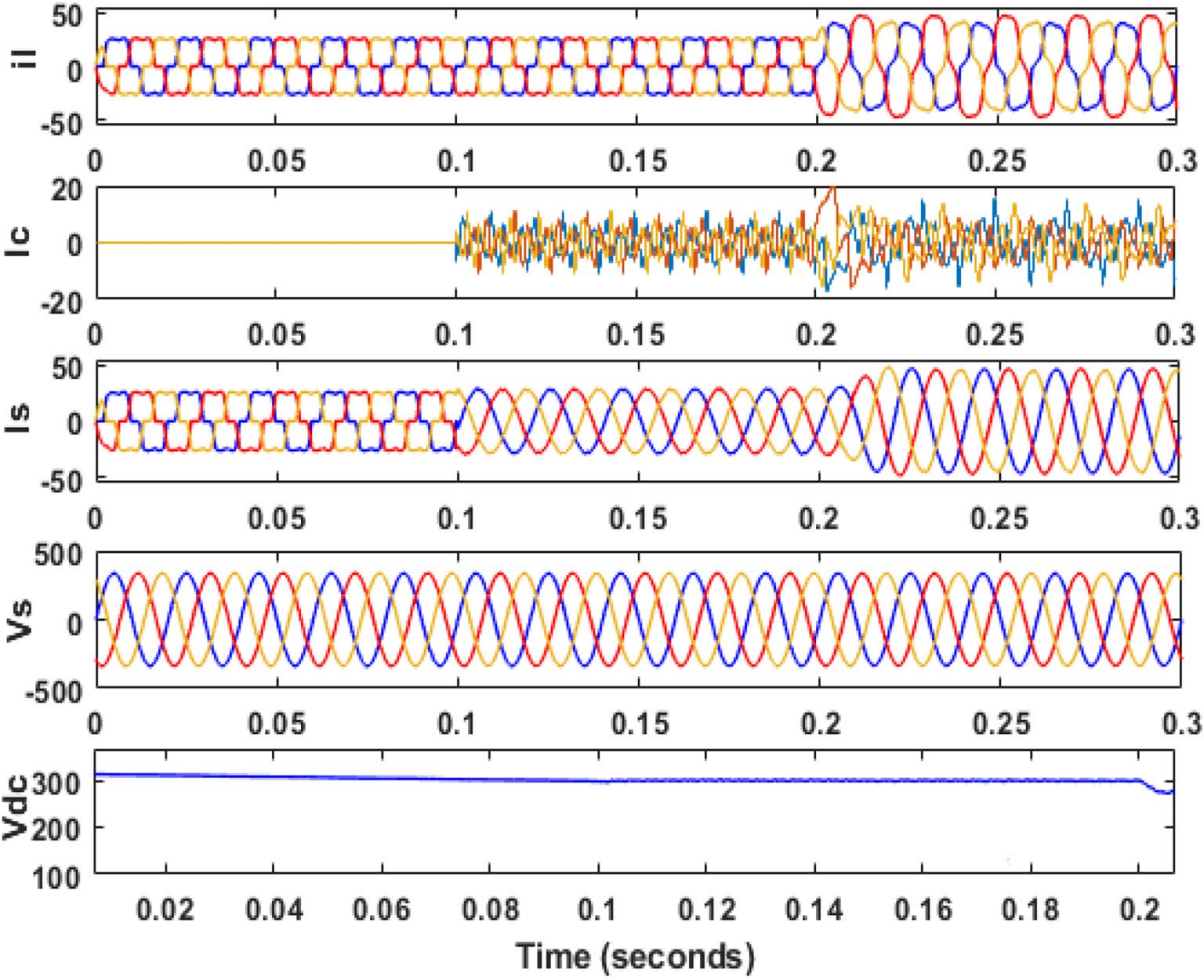
Performance characteristic of proposed SAPF with addition of an unbalanced three-phase load.

**Figure 17:**
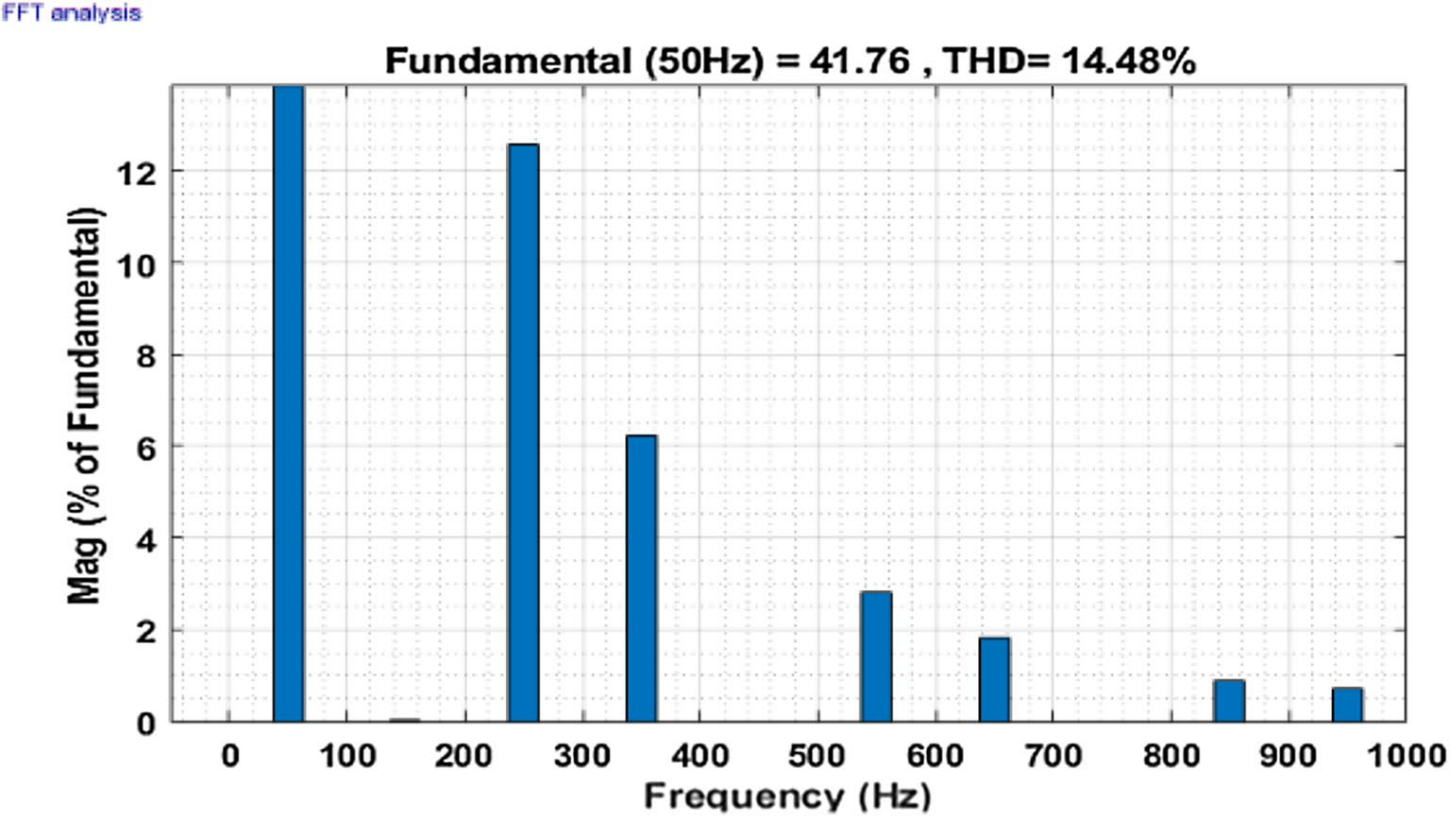
THD of grid current ‘$i_{s}$’ before compensation with addition of an unbalanced three-phase RL load.

**Figure 18:**
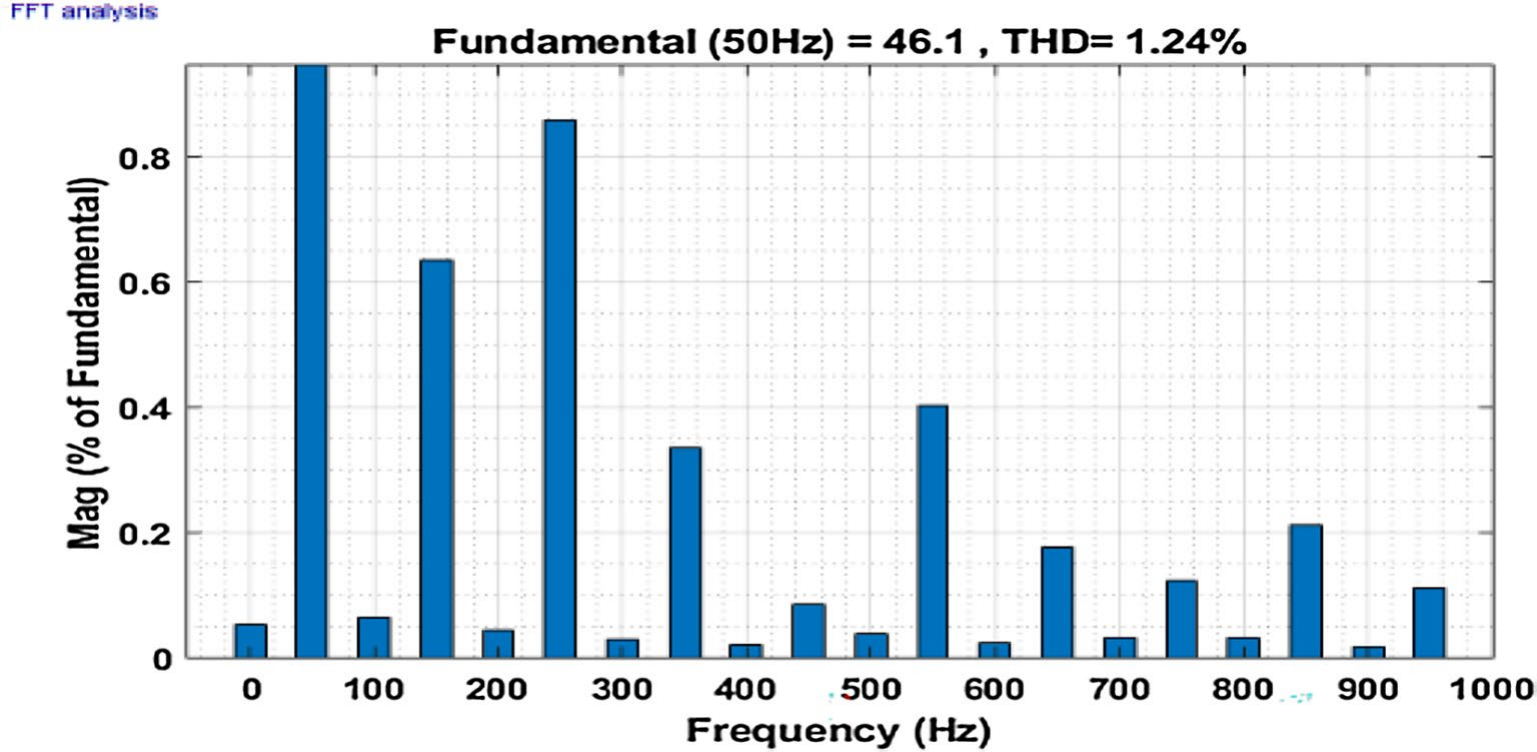
THD of grid current ‘$i_{s}$’ after compensation with addition of an unbalanced three-phase RL load.

Then the system is observed for an unbalanced loading condition, where an unbalanced three-phase inductive RL load is added parallel to the present non-linear load system. The SAPF is introduced at 0.1 s the unbalanced inductive load is added parallel to the previous load system at 0.2 s. The Fig. 16 shows the performance at this condition. Figures 17 and 18 show the FFT result for this unbalanced loading condition before and after initialization of filter respectively. The result shows the THD value of the grid source current without compensation is 14.48%, which minimized to 1.24% after compensation.

## Comparison with ($i_{d} - i_{q}$) and $i\cos \emptyset$ control techniques

Here the operating characteristic of the proposed SAPF with the NBP-based $i\cos \emptyset$ technique is compared with the other two control techniques. These are two conventional techniques ($i_{d} - i_{q}$ control technique and $i\cos \emptyset$ control technique). The observations are taken in each loading condition and compared with the proposed NBP-based $i\cos \emptyset$ technique. The comparison between these three control techniques for the SAPF is presented in Table 2.

**table 2:** Comparison of control techniques based on THD% in source current in different loading condition.

Control technique	Time invariant non-linear load		Step change in non-linear load		Addition of 1-phase RL load between phase ‘a’ and ‘b’		Addition of 3-phase RL imbalanced load	
	Before filtering	After filtering	Before filtering	After filtering	Before filtering	After filtering	Before filtering	After filtering
$i_{d} - i_{q}$	21.56%	4.91%	22.02%	4.97%	13.58%	4.25%	14.48%	4.78%
$i\cos \emptyset$	21.56%	3.86%	22.02%	3.95%	13.58%	3.19%	14.48%	3.52%
NBP-based $i\cos \emptyset$	21.56%	1.77%	22.02%	2.49%	13.58%	1.61%	14.48%	1.24%

### $i_{d} - i_{q}$ control technique

The performance of the SAPF using $i_{d} - i_{q}$ control technique is also examined. The THD of the load current $i_{l}$ is 21.56%, the THD of the grid source current $i_{s}$ is reduced to 4.91%. When a step change in load is done, it is observed that the THD value of source current is diminished from 22.02 to 4.97% after initiation of SAPF. When the single-phase RL load is connected in between phase ‘a’ and phase ‘b’ the THD value is reduced from 13.58 to 4.25%. In case of unbalanced three-phase inductive load, the THD value of source current comes down from 14.48 to 4.78% after filtration.

### $i\cos \emptyset$ control technique

The performance of the SAPF using $i\cos \emptyset$ control method is examined. The THD of the load current $i_{l}$ is 21.56%, and the THD of the grid source current $i_{s}$ is reduced to 3.86%. When a step change in load is done it is detected that the THD value of source current is diminished from 22.02 to 3.95% after compensation. When the single-phase RL load is connected in between the phases ‘a’ and ‘b’ the THD value is reduced from 13.58 to 3.19% in case of a three-phase unbalanced inductive load the THD value of source current comes down from 14.48 to 3.52% after filtration.

The source current harmonic elimination relies upon the active and reactive components of the load current. And, for PCC voltage balancing the DC-link capacitance–voltage management is important. The voltage management can be done by using the weights of the load components, which are extracted by an LPF. These weighted values are not updated in the classical methods like $i_{d} - i_{q}$ and $i\cos \emptyset$ control techniques. But these weights are updated in the NBP-based $i\cos \emptyset$ method. So, the voltage regulation becomes more effective. On the basis of the THD value of source current, the NBP-based $i\cos \emptyset$ technique is more effective for our proposed SAPF to eliminate the harmonics.

The DC-link capacitance voltage performance is also better in the case of the NBP-based $i\cos \emptyset$ method than in the other two methods. It is noticed that the SAPF with NBP-based $i\cos \emptyset$ gives results with less harmonics with reduced DC-link capacitance voltage. There are some other advantages such as reduced MLI rating, and low DC link capacitor stress. So, it is concluded that the SAPF operating with the NBP-based $i\cos \emptyset$ control technique is better than the conventional techniques.

## Real-time implementation and experimental result of the proposed SAPF

**Figure 19:**
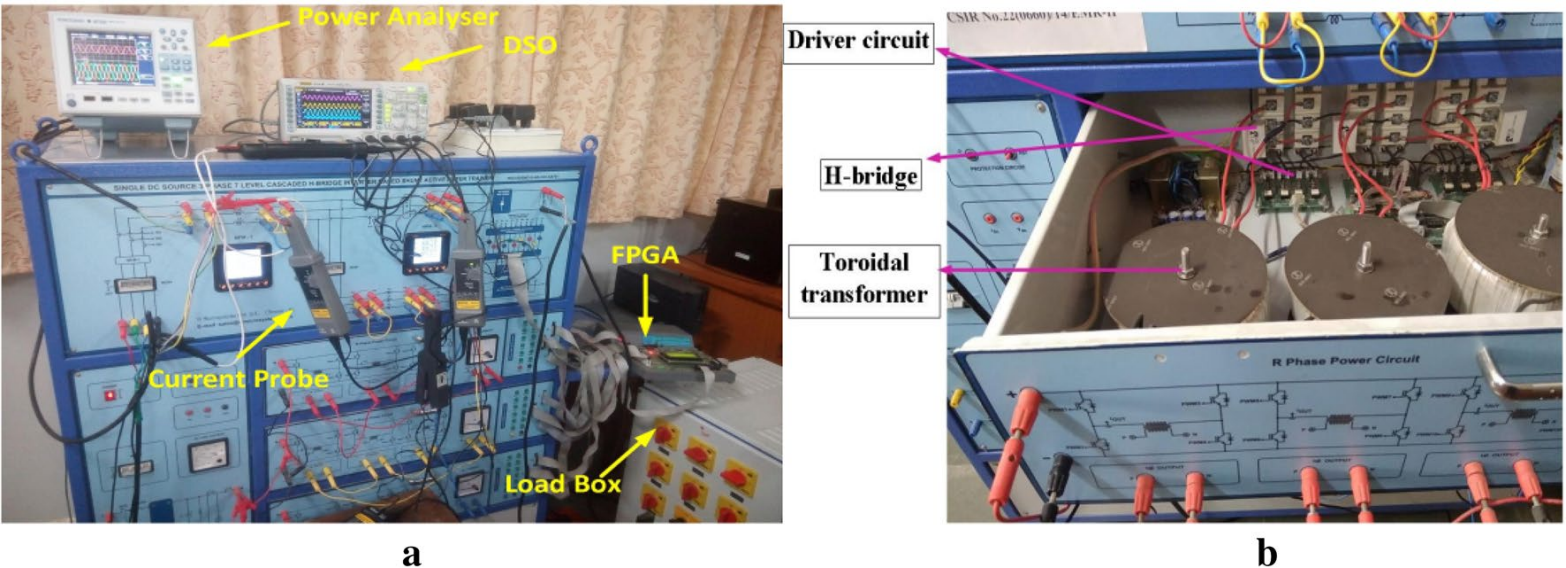
Experimental set up of the proposed system: (**a**) whole system, (**b)** connection of transformer in SAF for phase-A.

The implementation of the hardware of the suggested SAPF topology is presented in Fig. 19. The hardware arrangement consists of the suggested cascaded MLI incorporated of three modules. The modules are cascaded through a transformer with filter inductors connected in series. The setup also includes a single DC link capacitor, voltage sensor, current sensor, 3-phase uncontrolled rectifier bridge, FPGA module, etc.

**Figure 20:**
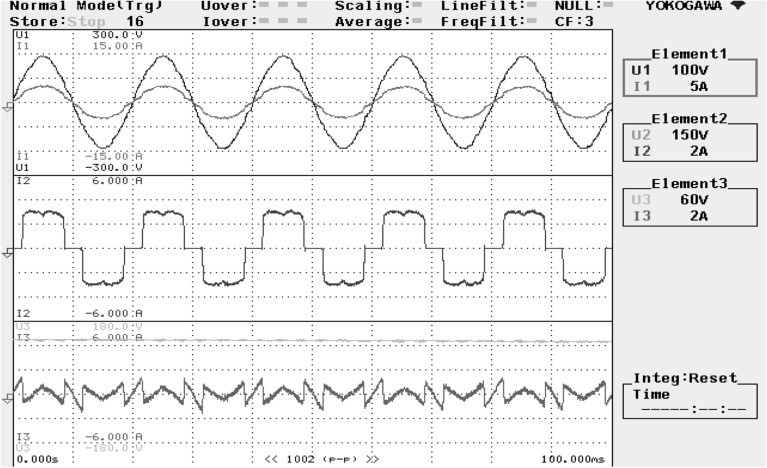
SAPF performance under steady state condition.

The reference DC-link capacitance voltage is predetermined at 160 V. For recording the experimental results, a power analyzer with six-channels is connected. The hardware arrangement is probed under balanced invariable source voltage condition. The SAPF operation is examined under balanced non-linear loading steady state circumstances is presented in Fig. 20. The supply voltage ‘$v_{s}$’, grid supply current ‘$i_{s}$’, load current ‘$i_{l}$’, and compensating current ‘$i_{c}$’ of a single phase is shown here.

**Figure 21:**
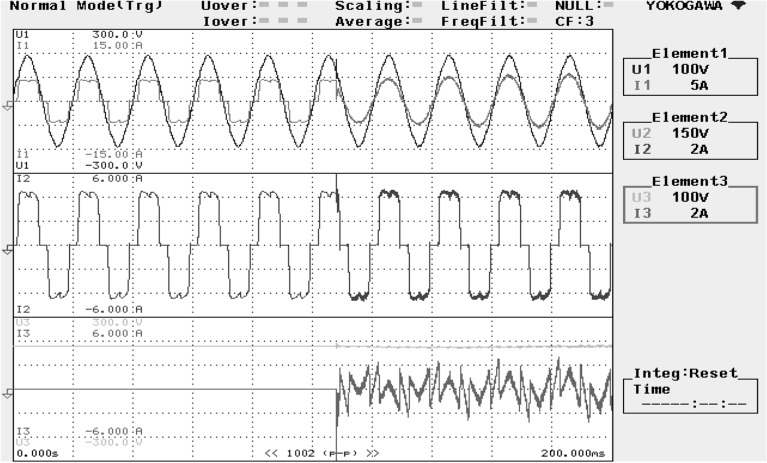
SAPF performance under transient condition.

**Figure 22:**
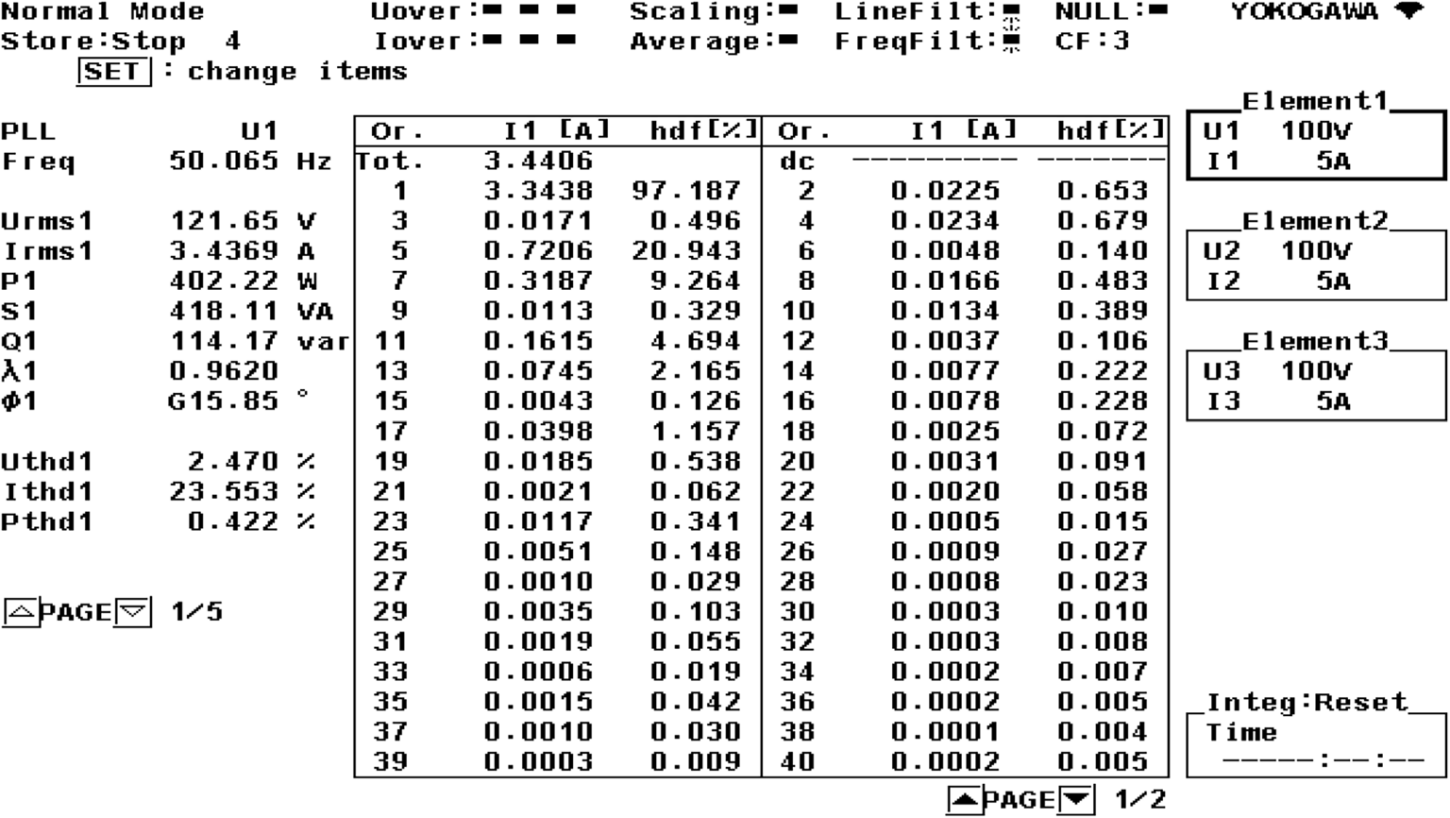
FFT result of the supply current under balanced load, before compensation.

**Figure 23:**
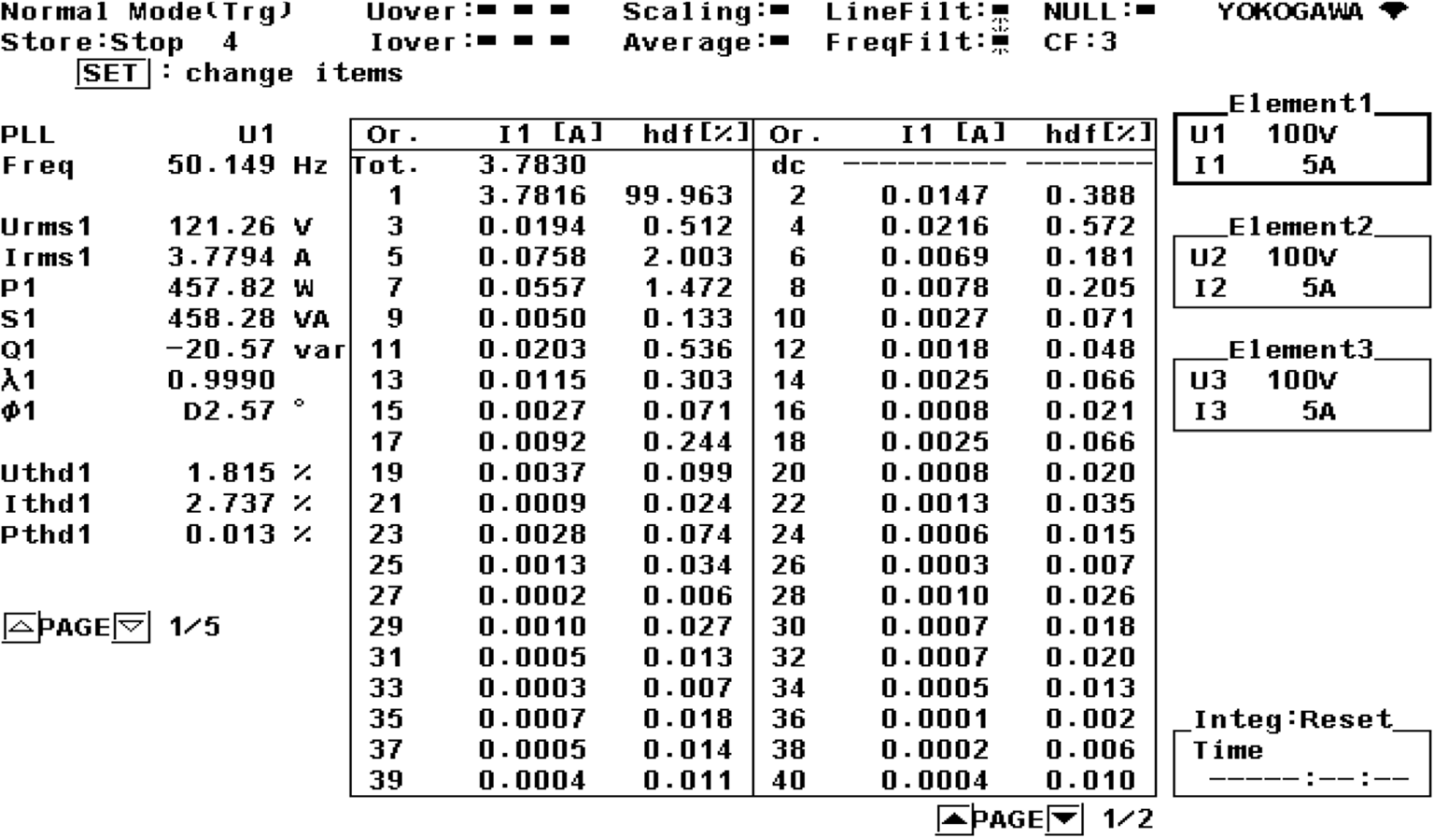
FFT result of the supply current under balanced load, after compensation.

**Figure 24:**
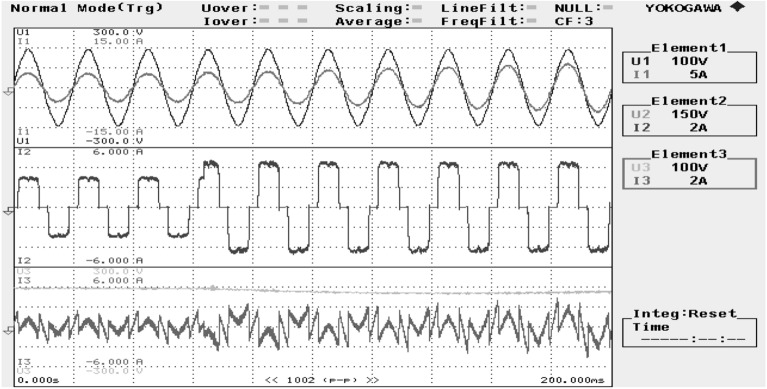
SAPF Performance under step variation in non-linear load.

**Figure 25:**
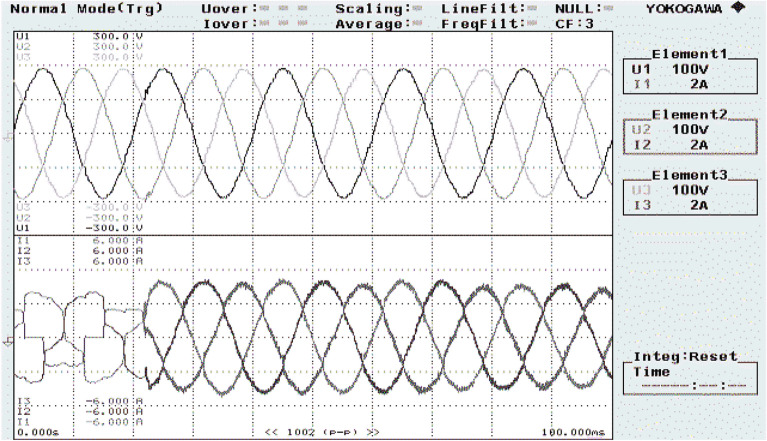
SAPF performance under unbalanced inductive load (single-phase RL load connected between phase ‘a’ and ‘b’ at PCC).

Figure 21 shows the operational behavior of the SAPF under the transient condition. Here the supply voltage ‘$v_{s}$’, supply current ‘$i_{s}$’, load current ‘$i_{l}$’, and compensating current ‘$i_{c}$’ before and after reimbursement is shown. From this it is clearly understood that the harmonics present in the supply current is removed and it turns into sinusoidal after filtration. This operation is done with a smooth and slight variation in DC link capacitance voltage. The FFT result of the working of the SAPF is presented in Figs. 22 and 23. These show that the THD of supply current under non-linear load in balanced condition, before compensation is 23.55%, and it reduced to 2.73% after compensation. Figure 24 shows the operational characteristic of the SAPF during a sudden step changed load. Here also the $v_{dc}$ varies smoothly to generate required compensating current and settles soon. The filter operates very efficiently at the transient. The performance under unbalanced loading condition (where a single-phase inductive (RL) load is connected between line ‘a’ and ‘b’ at PCC) is presented in Fig. 25. The filter seems to be very effective for unbalanced loading condition also.

**Figure 26:**
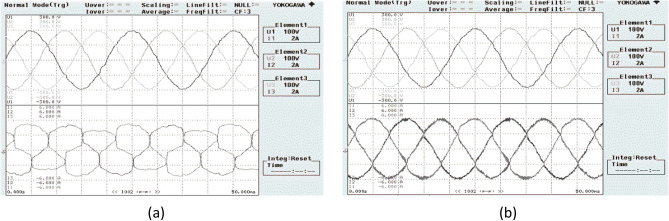
SAPF Performance under unbalanced load (three-phase load connected at PCC). (**a**) Before compensation and (**b**) after compensation.

**Figure 27:**
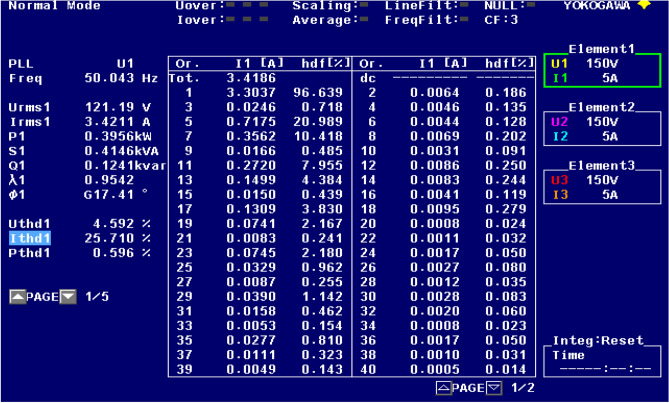
FFT result of the source current under unbalanced load, before compensation.

**Figure 28:**
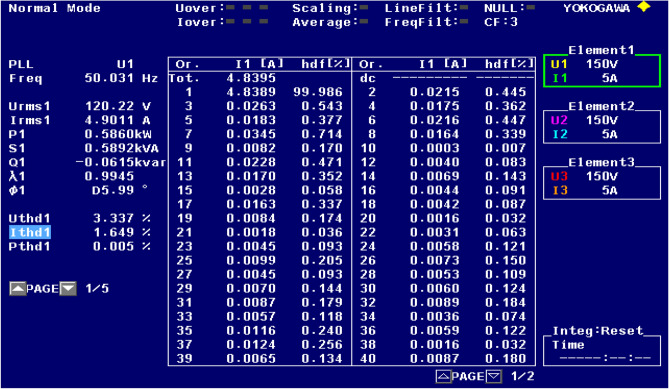
FFT result of the source current under unbalanced load, before compensation.

The performance characteristic of SAPF under unbalanced load (where a three-phase unbalanced inductive load is connected in parallel at the PCC) is presented in Fig. 26. Where (a) shows the characteristics before compensation and (b) shows the characteristic after compensation. Figures 27 and 28 represent the FFT result of the source current under unbalanced load, where it is found that the THD of supply current is 25.71% before compensation, and it is reduced to 1.65% after compensation.

## Conclusion

In this paper the effectiveness of proposed NBP-based $i\cos \emptyset$ technique is compared with another two classical control techniques for power quality improvement on cascaded MLI based shunt active power filter. The harmonic current removal by the recommended control technique found to be more accurate compared to other control techniques discussed in the literature. The reference compensating current generation is done by using NBP-based $i\cos \emptyset$ method. As this control technique is having a feedback system, it can produce a precise weighting factor. So, this controller is very effective to reduce harmonics with less amount of voltage variation at DC link capacitor. From the simulation and experimental hardware result it is clear that, the recommended technique is more efficient than $i_{d} - i_{q}$ method and $i\cos \emptyset$ method to eliminate the harmonics in balanced and unbalanced non-linear loading condition.

## Data Availability

The datasets used and/or analysed during the current study available from the corresponding author on reasonable request.
